# Early Intervention in Psychosis and Management of First Episode Psychosis in Low- and Lower-Middle-Income Countries: A Systematic Review

**DOI:** 10.1093/schbul/sbae025

**Published:** 2024-03-25

**Authors:** Saeed Farooq, Nishani Fonseka, Malik Wajid Ali, Abbie Milner, Shumaila Hamid, Saima Sheikh, Muhammad Firaz Khan, Mian Mukhtar-ul-Haq Azeemi, Gayan Ariyadasa, Abdul Jalil Khan, Muhammad Ayub

**Affiliations:** School of Medicine, Faculty of Medicine and Health Sciences, Keele University, Staffordshire, UK; Research and Innovation Department, Midlands Partnership NHS Foundation Trust, St George’s Hospital, Stafford, UK; School of Medicine, Faculty of Medicine and Health Sciences, Keele University, Staffordshire, UK; Armed Forces, Institute of Mental Health, Rawalpindi, Pakistan; School of Medicine, Faculty of Medicine and Health Sciences, Keele University, Staffordshire, UK; Public Health Department, Institute of Public Health & Social Sciences, Khyber Medical University, Peshawar, Pakistan; School of Medicine, Faculty of Medicine and Health Sciences, Keele University, Staffordshire, UK; Institute of Mental Health & Behavioral Sciences, Khyber Medical University, Peshawar, Pakistan; Department of Psychiatry, Medical Teaching Institution, Lady Reading Hospital, Peshawar, Pakistan; School of Medicine, Faculty of Medicine and Health Sciences, Keele University, Staffordshire, UK; Department of Family Medicine, Institute of Public Health and Social Sciences, Khyber Medical University, Peshawar, Pakistan; Department of Psychiatry, Queen’s University, Kingston, Canada; Primary Department of Psychiatry, University College London, London

**Keywords:** psychiatry, first-episode psychosis, early intervention in psychosis services, pharmacological intervention, non-pharmacological interventions

## Abstract

**Background and Hypothesis:**

People with first-episode psychosis (FEP) in low- and lower-middle-income countries (LMIC) experience delays in receiving treatment, resulting in poorer outcomes and higher mortality. There is robust evidence for effective and cost-effective early intervention in psychosis (EIP) services for FEP, but the evidence for EIP in LMIC has not been reviewed. We aim to review the evidence on early intervention for the management of FEP in LMIC.

**Study Design:**

We searched 4 electronic databases (Medline, Embase, PsycINFO, and CINAHL) to identify studies describing EIP services and interventions to treat FEP in LMIC published from 1980 onward. The bibliography of relevant articles was hand-searched. Preferred Reporting Items for Systematic Reviews and Meta-Analyses (PRISMA) guidelines were followed.

**Study Results:**

The search strategy produced 5074 records; we included 18 studies with 2294 participants from 6 LMIC countries. Thirteen studies (1553 participants) described different approaches for EIP. Pharmacological intervention studies (*n* = 4; 433 participants) found a high prevalence of metabolic syndrome among FEP receiving antipsychotics (*P* ≤ .005). One study found a better quality of life in patients using injectables compared to oral antipsychotics (*P* = .023). Among the non-pharmacological interventions (*n* = 3; 308 participants), SMS reminders improved treatment engagement (OR = 1.80, CI = 1.02–3.19). The methodological quality of studies evidence was relatively low.

**Conclusions:**

The limited evidence showed that EIP can be provided in LMIC with adaptations for cultural factors and limited resources. Adaptations included collaboration with traditional healers, involving nonspecialist healthcare professionals, using mobile technology, considering the optimum use of long-acting antipsychotics, and monitoring antipsychotic side effects.

## Introduction

The initial 1–3-year period after the onset of psychosis is considered as the critical period in the outcome of first-episode psychosis (FEP).^1^ The lack of treatment during this period sets in motion the neuropathological and neurochemical processes which result in poorer outcomes. Individuals experiencing FEP often have a decline in their social functioning alongside negative symptoms like a lack of motivation and reduced ability to experience pleasure. Social functioning tends to diminish as individuals face challenges in maintaining relationships, engaging in daily activities, and fulfilling societal roles.

The duration of untreated psychosis (DUP) refers to the period between the onset of the initial psychotic symptom and the commencement of appropriate treatment with antipsychotic medication. The long DUP before appropriate treatment is associated with worse outcomes in overall functioning, symptom severity, and quality of life, as well as lower levels of recovery from the first psychotic episode.^2,3^

The robust evidence for the deleterious effect of untreated or poorly treated FEP during the initial years of FEP led to the development of early intervention in psychosis (EIP) services in many countries. EIP services aim to detect and manage psychotic symptoms and associated psychological and behavioral effects of FEP during the critical first 3 years after the onset of psychosis.^4^ These services vary between countries but often share similar components such as explicit admission criteria, small patient-to-staff ratios, and evidence-based pharmacological and psychosocial treatments.

EIP services are now a widespread therapeutic approach across Europe, United States, and Australasia. Recent systematic reviews and meta-analyses show that EIP services are both effective and cost-effective.^5–8^ However, almost all evidence in these systematic reviews comes from high-income countries (HIC).

According to the United Nations Population Fund, 90% of young people across the world live in LMIC.^9^ Those young people experiencing FEP within LMIC experience a prolonged DUP of approximately 125 weeks compared to 62.5 weeks in HIC.^10^ Such a long period of untreated psychosis is associated with poor response to treatment, greater disability, and probably increased mortality.^11^ Therefore, understanding the status and effectiveness of EIP services in LMIC is crucial to inform the development of treatment pathways for FEP and improve clinical outcomes in young people with FEP.

There is a paucity of literature on the effectiveness of pharmacological and psychosocial interventions for treating FEP that can be used in EIP services in LMIC. Resource constraints and important cultural differences mean that commonly used interventions such as cognitive behavioral therapy (CBT), which are readily available in EIP services in HIC, cannot be used in LMIC.^12^ There are also important differences in access to pharmacological treatments, the pharmacokinetics of antipsychotic treatments, and the greater propensity for metabolic side effects of commonly used antipsychotics for large populations in many LMIC.^13^

Many patients with FEP in LMIC present to traditional and spiritual healers (TSH) as their first care providers,^14^ and this must be considered in care pathways for providing EIP services in LMIC. Therefore, a “one-size-fits-all” approach for EIP cannot be utilized and it is important to examine the provision of EIP services and the interventions to treat FEP in LMIC.

To the best of our knowledge, there is currently no systematic review that examines the evidence on the configuration of EIP services or their effectiveness in treating FEP in LMIC. We aim to systematically review the literature on the components and configuration of EIP services in LMIC and the interventions that can be used in these services to treat FEP in LMIC settings. Specifically, this study will address the following questions:

1. What is the existing situation of EIP services in LMIC, including care pathways for assessment and management of FEP, the characteristics of EIS, and the configuration of services?
2. Is EIP effective or cost-effective in improving the outcomes for people with early psychosis in LMIC?
3. What is the evidence for the effectiveness of pharmacological and non-pharmacological interventions that have been studied in the context of LMIC, and can they be used in EIP services in these countries?

## Methods

We followed the PRISMA guidelines^15^ (supplementary appendix 1) for evidence synthesis and the protocol was registered prior to starting the study (PROSPERO 2022 CRD42022338379).

### Inclusion and Exclusion Criteria

We included the studies reporting the effectiveness of pharmacological or psychosocial intervention among people with FEP, and studies describing the EIP services in any setting from LMIC (supplimemtary appedix 2). We limited the search to 1980 onwards as the early intervention services and interventions for treating and evaluating focused specifically on FEP only started in 1990 onwards. As some interventions used in FEP might have commenced before 1990, the search criteria were set to cover studies from 1980 onwards.

EIP is mostly limited to the 15–35 age group in HIC.^16^ In view of the sparse literature, we decided to include EIP for FEP regardless of age in LMIC.

### Definitions of Key Concepts

#### FEP.

The clinical definition for FEP described by the NICE guidelines is the first time a person experiences a combination of symptoms known as psychosis.^16^ The definitions of “first episode psychosis” in the literature vary. FEP is usually defined as psychosis (meeting ICD or DSM criteria for the psychotic disorder) in people who have not received regular antipsychotic treatment for more than 6 weeks prior to contact with the index service.^17^ We included studies irrespective of the definition used and we provide the definition of FEP as employed by different studies (supplementary appendix 3). We also included studies describing the at-risk mental state as these states commonly precede FEP.^16^

#### EIP Services.

EIP services are defined in terms of providing a set of services for people with FEP during the first 2–5 years after contact with services. We included the studies describing EIP in any form or scope. Studies describing the early identification of people at high risk were also included.

#### Pharmacological and Non-pharmacological Interventions.

Any drug treatment used for treating FEP was considered a pharmacological intervention. The non-pharmacological interventions included psychosocial interventions such as CBT, relaxation exercises, occupational therapy, family psychoeducation, and individual psychoeducation.

### Search Strategy

Four databases were electronically searched: CINAHL, EMBASE, MEDLINE, and PsycINFO. The preliminary search was carried out to refine the search terms. The search strategy was developed in consultation with the systematic review team that included psychiatrists with extensive experience in EIP, psychologists, and an information specialist. The search strategy was developed with a focus on the review questions, types of studies, databases, Mesh terms, variations of search terms using parentheses and Boolean operators, and insights gained from previous similar literature searches. Each low- and middle-income country was searched separately.

The detailed search strategy and search terms used are given in supplementary appendix 4 and the following terms were included in the search strategy: “psychosis,” “psychotic,” “early intervention,” “FEP (first episode intervention),” Schizophrenia, schizophrenia spectrum and other psychotic disorders, early intervention schizophrenia “PLE (psychosis-like illness),” “ultra high-risk mental state,” “early medical intervention,” “health plan implementation,” “at-risk mental state,” “prodrome,” “early intervention psychosis services,” “care,” and “care pathways.” The search strategy was last updated in June 2023. The citations and bibliographies of the full-text articles retrieved from the search were hand-searched to avoid duplication and missing important key articles. Also, a secondary hand search of references was carried out to identify any additional articles relevant to the review.

### Study Selection Process

The titles and abstracts of the articles were independently screened by 2 reviewers (S.S. and M.W.). Any disagreement about an article at this stage was discussed with a third reviewer (S.F.) to reach a consensus. The full texts of the included studies that met the inclusion criteria were assessed independently by 2 reviewers (N.F. and A.M.), and any differences were subsequently discussed with a third reviewer (S.F.).

### Data Extraction

The data were extracted independently from each of the eligible articles by 2 reviewers (A.M. and S.H.) using an Excel form created especially for this review. In addition to the study characteristics, the data was extracted on the following key variables: (a) intervention studies: drug therapy, type of non-pharmacological intervention (psychological, family education), setting, control group, components, followed-up period, outcome of effectiveness (symptoms, functioning or other parameters). (b) EIP services studies: location, components, service providers/facilitators, effectiveness as a primary outcome, and implementation strategies, etc., as secondary outcomes.

### Data Synthesis

We planned to do a meta-analysis of included studies for key outcomes. However, on examining the included studies, it was observed that the studies varied in terms of the type of interventions/services, methods used, and outcomes considered. Therefore, it was not appropriate to do a meta-analysis, and synthesis without meta-analysis was performed following the guidelines.^18^ These guidelines outline 9 essential categories of information that must be provided in the instance that a quantitative synthesis is not feasible (supplementary appendix 5). This synthesis incorporated the country in which the research was carried out, details of interventions and key findings, characteristics of EIP services in LMIC, and proposed outcomes.

### Risk of Bias Assessment

The quality of the studies was assessed independently by 2 reviewers (N.F. and G.A.) using checklists for different study designs. Cochrane collaboration tool^19^ was used for randomized controlled trials (RCTs) and Risk of Bias in Non-Randomized Studies of Interventions^20^ was used for quasi-experimental studies. STrengthening the Reporting of OBservational studies in Epidemiology (STROBE) was used for observational studies (supplementary appendix 6).^21^ The qualitative study was screened as a part of the overall literature results using Joanna Briggs Institute critical appraisal checklist criteria (supplementary appendix 6).

### Patient and Public Involvement

Patient and public involvement and engagement (PPIE) for this review is part of the THEHOPE study, as described in the study’s protocol.^22^ THEHOPE is a research program that aims to develop a collaborative approach with TSH for the early detection and management of FEP in Pakistan. PPIE has been crucial at every stage of THEHOPE, including the design and conduct of this systematic review. The members of patients and the public who formed the PPIE group in Pakistan identified the need for developing evidence-based guidance for the management of FEP in Pakistan, which led to the choice of outcome measures and search strategy for this review. At each stage, PPIE and community service user groups from the University of Keele and Peshawar, Pakistan have provided key suggestions for the study. The priorities, experiences, and preferences of these groups will inform the dissemination of findings from this review.

## Results

**Fig. 1.**
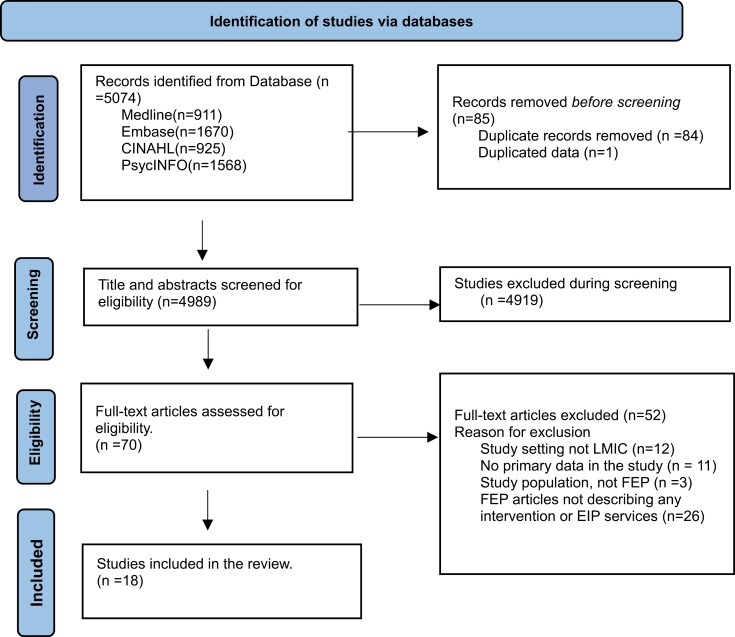
PRISMA flow chart of included studies.

The search strategy produced 5074 records, of which 18 studies met the inclusion criteria. The PRISMA flow diagram (figure 1) illustrates the searches, screenings, and selection outcomes. The studies included articles describing EIP services in LMIC (*n* = 6, 33.3%), pharmacological interventional (*n* = 3, 16.7%), non-pharmacological interventional (*n* = 4, 22.2%), and both pharmacological and non-pharmacological (*n* = 5, 27.8%) for FEP.

### Study Characteristics

**Fig. 2.**
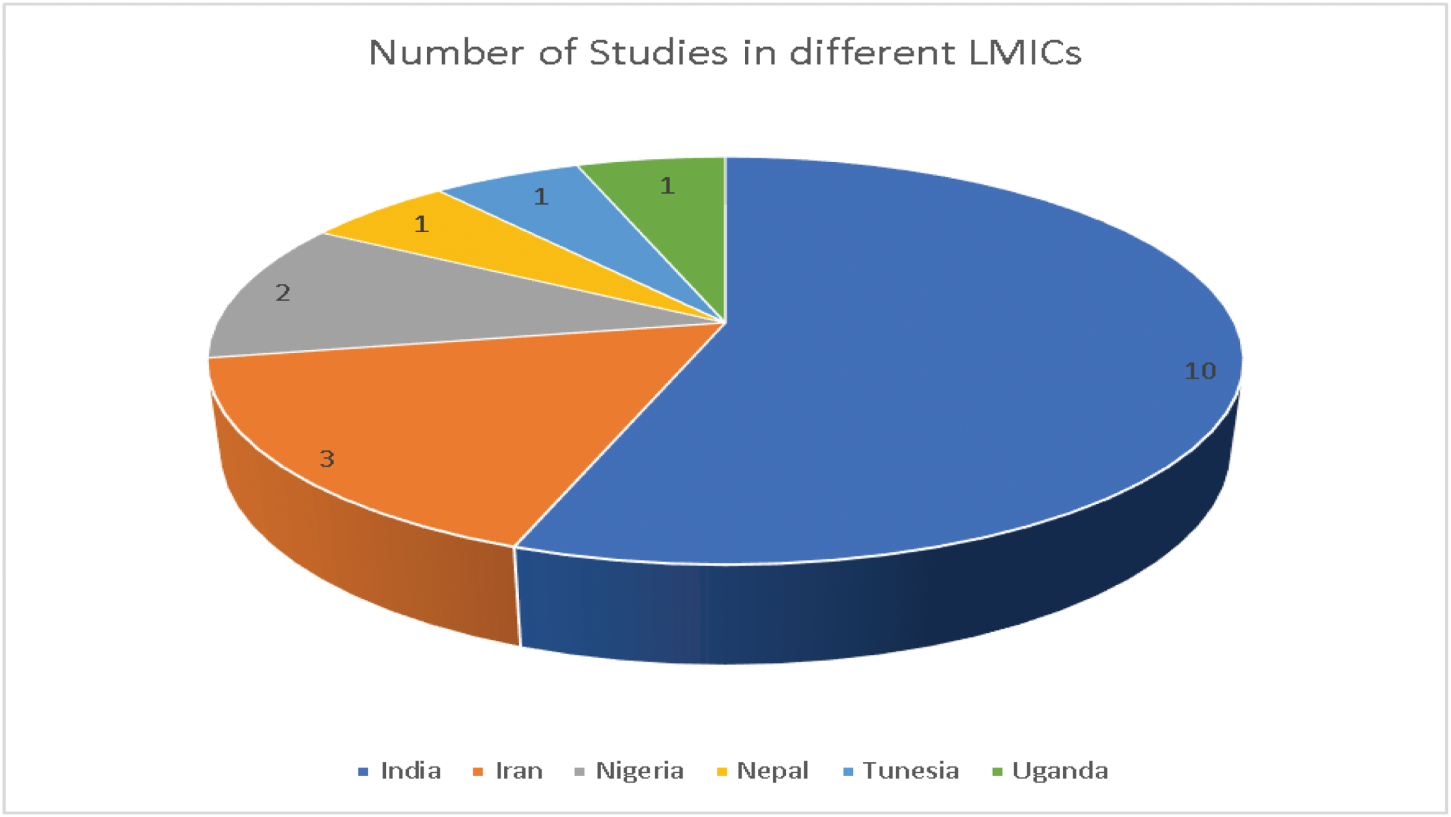
Different LMIC of included studies.

The characteristics of the studies are listed in table 1. The included studies were conducted in hospital settings between 2008 and 2023 in the following 6 countries (figure 2): India (*n* = 10), Iran (*n* = 3), Nigeria (*n* = 2), Nepal (*n* = 1), Tunisia (*n* = 1), and Uganda (*n* = 1).

**Table 1.** Characteristics of the included studies

Non-pharmacological interventions (psychological, social, and psychosocial)					
Author, year, country	Outcomes of interest	Intervention framework and comparator (if any)	Study design	Sample size	Demographic characteristics (mean age and percentage of females)
Sadath et al.^23^ India	Expressed emotions and social support for carers of persons with FEP	Group intervention TAU	Quasi-experimental nonequivalent comparison group design	Intervention: *N* = 37 TAU: *N* = 34	Intervention: 47.43 (Carer); 23.87 (Patient) TAU 46.89 (Carer); 24.25 (Patient) Carer (ALL): *F* = 37.3%
Adhikari^24^ Nepal	Efficacy of ECT in patients with FES	ECT ECT non-receivers	Prospective Study	*N* = 45 ECT received = 12 ECT not received 33	<20: *n* = 3, 20–29: *n* = 31, 30–39: *n* = 9, >40: *n* = 2 *F* = 26.7%
Thomas et al.^25^ Nigeria	Effectiveness of SMS reminders of clinic appointments among FEP patients	Appointment date card and SMS text reminder Appointment dates on the card	RCT	*N* = 192 Intervention: *N* = 95 Control N:97	33.76 (±11.9 y) Intervention: *F* = 47% Control: *F* = 61%
Pharmacological interventions					
Author, year, country	Outcomes of interest	Intervention framework and comparator (if any)	Study methods/design	Sample size	Demographic characteristics (mean age and percentage of females)
Saddichha et al.^13^ India	Effects of olanzapine, risperidone, and haloperidol on the development of metabolic syndrome in FEP patients	Administration of 1 of the 3 drugs-olanzapine, risperidone, or haloperidol Healthy controls (no medication used)	Randomized double-blind control study	*N* = 150 Intervention: *N* = 99 Control: *N* = 51	Intervention 26.0 ± 5.5 *F* = 47.5% Control 27.5 ± 5.9 *F* = 41.2%
Tabatabaee et al.^26^ Iran	Acute treatment response and its predictors in FEP patients	Treatment with medications as prescribed by the treating psychiatrists. N/A	Naturalistic study—pre and post-assessments	*N* = 163	26.3 y (SD 9.9) *F* = 44.2%
Modabbernia et al.^27^ Iran	Efficacy of melatonin in the prevention of olanzapine-induced metabolic side effects	Melatonin 3 mg/d Placebo	Randomized, double-blind, placebo-controlled, parallel-group study	*N* = 48 Melatonin *N* = 24 Placebo: *N* = 24	Melatonin group: 32.7 (7.3). *F* = 28% Placebo group: 32.8 (8.2), *F* = 33%
Kaur et al.^28^ India	Effectiveness of LAI antipsychotics for FES over oral antipsychotics	LAI haloperidol Oral haloperidol	RCT	*N* = 72 LAI: *N* = 38 Oral: *N* = 34	All: 29 y LAI: *F* = 42.1% Oral: *F* = 32.4%
Both non-pharmacological and pharmacological studies					
Author, year, country	Outcomes of interest	Intervention framework and comparator (if any)	Study methods/design	Sample size	Demographic characteristics (mean age and percentage of females)
Malla et al.^29^ India (compared with Canada)	Clinical outcomes in FEP treated in Chennai (LMIC) and Montreal (HIC) using a similar EIP treatment protocol	Protocols for treatment of FEP	Longitudinal, 2-y prospective outcomes study Pre- and post-assessment	Chennai: *N* = 168 Canada: *N* = 165	Chennai: 26.60 (SD 5.24) *F* = 51.2% Montreal: 24.20 (SD 5.3) *F* = 32.7%
Ventura et al.^30^ Tunisia	Feasibility of developing a CHiRP program to identify youth at high clinical risk	CHiRP N/A	A pilot feasibility study	*N* = 10 CHR+: *N* = 6 CHR−: *N* = 4	Average age 19.8 (SD 5) *F* = 30%
Chiliza et al.^31^ Nigeria and South Africa	Feasibility and effectiveness of depot antipsychotic combined with an AMP in first-episode schizophrenia	Depot antipsychotic (flupenthixol decanoate) and AMP N/A	Exploratory, noncomparative study Pre- and post-assessment	All: *N* = 207 (81 in Nigeria, 126 from South Africa)	25.87 (SD 6.92) F = 34%
Iyer et al.^32^ India (compared with Canada)	Differences in patients’ and families’ service engagement in similarly structured first-episode psychosis programs in Montreal, Canada, and Chennai	FEP program N/A	Prospective study Pre- and post-assessment	*N* = 333 Chennai: *N* = 168 Montreal: *N* = 165	Chennai: 26.60 (SD 5.24) *F* = 51% Montreal: 24.20 (SD 5.3) *F* = 33%
Rangaswamy et al.^33^ India	The course and outcome of persons with the untreated first episode of psychosis, factors affecting the outcome, and DUP and its impact on outcome in psychosis.	First episode psychoses program N/A	Prospective study (pilot study) Pre- and post-assessment	*N* = 47	29.7 *F* = 70.2%
EIP services					
Author, year, country	Outcomes of interest	Type of service	Study methods/design	Sample size	Demographic characteristics (mean age and percentage of females)
Iyer et al.^34^ India (compared with Canada)	One-year clinical and functional outcomes of FEP program between Chennai and Montreal	First episode psychoses program	Prospective study	India: *N* = 61 Montreal: *N* = 88	India: 29.26 (10.95) *F* = 62.3% Montreal: 22.74 (3.94) *F* = 32.95%
Singh et al.^35^ India	Comparison of pathways to care in FEP between North and South India, to inform solutions to bridge the treatment gap	FEP care program	Cross-sectional observation study	*N* = 177 South: *N* = 72 North: *N* = 105	South: 28.27 (7.80) *F* = 54.2% North: 27.15 (7.81) *F* = 41.9%
Mwesiga et al.^36^ Uganda	Quality of the individual and group-level interventions in FEP	Pharmacotherapy, individual and family psychoeducation, vocational plan, etc.	Retrospective chart review	*N* = 156	Median age: 27 y *F* = 55.3%
Mottaghipour et al.^37^ Iran	Effectiveness of training health professionals in adherence to protocol	Training program in family psychoeducation	Evaluation of a training program-descriptive analysis	8 professionals	Not available
Vaitheswaran et al.^38^ India	Challenges in FEP intervention program in a specialist mental health facility using the Consolidated Framework for Implementation Research.	Pharmacotherapy, psychoeducation, supportive counseling, crisis management, stress management, caregiver support, medication adherence, relapse prevention, and monitoring for the side effects of medicines	Qualitative study-focus group/in-depth interviews	27 (15 patients with FEP and 12 caregivers)	25.6 y (SD 7.5) *F* = not available
Iyer et al.^39^ India	Development of PREMs and FREMs for application in early psychosis services research	Show me you care (PREM and FREM)—A patient- and family-reported measure of care experiences in early psychosis services	Development of tool and psychometric properties of a new measure	Chennai Patient: *N* = 29 Family: *N* = 27 Montreal Patient: *N* = 31 Family: *N* = 31	Patient sample Chennai: 26.31 (5.10) *F* = 48.28% Montreal: 24.00 (5.22) *F* = 38.7% Family sample Chennai: 21–60 y *F* = 51.85% Montreal: 21–70 y *F* = 80.65%

*Note*: AMP, assertive monitoring program; CHirP, clinical high-risk program; ECT, electroconvulsive therapy; FEP, first-episode psychosis; FES, first-episode schizophrenia; FREMs, family-reported experience measures; LAI, long-acting injectable; PREMs, patient-reported experience measures; RCT, randomized controlled trial; SMS, short message service; TAU, treatment as usual.

The 16 quantitative studies included the following: RCT (*n* = 4), quasi-experimental (*n* = 1), and observational studies (*n* = 11). The total number of participants in all included studies was 2294 and the mean sample size was 127.4. The mean age of the study sample was 29.05 (95% CI: 24.06–33.5).

### Studies on Pharmacological and Non-pharmacological Interventions in FEP

The brief descriptions of interventions and key findings are summarized in table 2 (supplementary appendix 7), with detailed outcome measures used in these studies given in table 3 (supplementary appendix 8). Pharmacological studies included 3 RCT and 1 longitudinal observational study. Saddichha et al.^13^ evaluated the effects of olanzapine (*n* = 35), risperidone (*n* = 33), and haloperidol (*n* = 31) on the development of metabolic syndrome (MetS) in an RCT and found that olanzapine was associated with the highest prevalence rates (20%–25%) of MetS compared to risperidone (9%–24%) and haloperidol (0%–3%). The overall prevalence was 5 times greater than the prevalence observed (*P* = .005 and *P ≤* .001) in the control group (*n* = 51). In a randomized, double-blind, placebo-controlled study, Modabbernia et al.^27^ examined the short-term effect of melatonin in the prevention of drug-induced (olanzapine) MetS. By the eighth week of the trial, individuals who were administered melatonin (*n* = 24) demonstrated a significantly lower increase in weight (*P* = .023), body mass index (*P* = .024), and waist circumference (*P* = .041), as compared to the placebo group (*n* = 24). Furthermore, the patients in the melatonin group showed a significant reduction in the PANSS total score (*P* = .014).

Kaur et al.^28^ compared the effectiveness of long-acting injectable (LAI) antipsychotics vs oral antipsychotic drugs. Patients (*n* = 72) with FEP (DSM-5) were randomly assigned to receive either oral haloperidol (*n* = 34) or LAI haloperidol (*n* = 38) for a duration of 12 weeks. Both groups showed a significant decrease in PANSS scores, while those on the LAI haloperidol demonstrated a significantly higher quality of life (*P* = .023). Tabatabaee et al.^26^ assessed the predictors of acute treatment response to antipsychotics in FEP patients (*n* = 163) in an inpatient unit in Iran. At the end of 5–7 weeks of follow-up, it was observed that the higher premorbid, lower baseline functioning, and acute onset were found to be the predictors of functional response (*x*^2^ = 30.093; df = 3; *P* ≤ .001; Nagelkerke *R*^2^ = 0.379).

Amongst non-pharmacological studies, Thomas et al.^25^ examined the effectiveness of short message service (SMS) reminders on clinical appointments in an RCT and found that receiving SMS helped to improve clinic attendance (OR = 1.80, 95% CI = 1.02–3.19). In a quasi-experimental study, Sadath et al.^23^ evaluated the effectiveness of group intervention (*n* = 37) vs treatment as usual (TAU) (*n* = 34) on expressed emotions (EE) and social support for carers of persons with FEP. Group intervention reduced EE significantly (*P* < .007) at the one-month follow-up, but the observation was not significant at the third month. The effectiveness of electroconvulsive therapy (ECT) in patients with FEP was assessed using a naturalistic study methodology and those who received ECT (*n* = 12) showed significant improvement in psychopathology (*P* = .001) and day-to-day functioning (*P* = .003) when compared to the group that did not receive ECT (*n* = 33) at 1-year post-ECT (Adhikari^24^).

### EIP Services in LMIC

Detailed findings from these studies are given in table 2 (supplementary appendix 7) and the brief findings are described here. Four studies used pre- and post-design. Three studies examined the effectiveness of EIP by comparing services in Canada and India.^29,32,34^ Both sites adopted similar protocols for the treatment of FEP, case management, individual and family intervention, psychoeducation, and CBT using key outcomes such as positive and negative symptoms, family function, and medication adherence. These studies conducted a comparative analysis of the FEP program in Canada and India. Significant improvement in positive (*P* ≤ .001) and negative symptoms (*P* ≤ .03) over the 24 months was observed in both the sites, Chennai (*n* = 168) and Montreal (*n* = 165). Family support was higher in Chennai (µ = 10.3 SD = 3.41) than in Montreal (µ = 8.79 SD = 3.46).^29^ The predictors of service disengagement were examined.^34^ Improved communication between the families and the treating teams (HR = 0.84, 95% CI 0.78–0.92), age at entry (HR = 0.89, 95% CI 0.80–0.98), and medication adherence (HR = 0.28, 95% CI 0.12–0.68) were found to be independently associated with reduced likelihood of service disengagement. Rangaswamy et al.^33^ assessed DUP and its impact on outcomes in patients (*n* = 47) with FEP and found that at the end of 2 years, the group that experienced remission (*n* = 28) had a substantially lower DUP compared to the group that remained consistently unwell (*n* = 10) (*t* = 4.048, sig = 0.002). The mean DUP for the remitted group was 11.40 months, whereas the mean DUP for the continuously ill group was 56.50 months. Authors commented that EIP is feasible even in environments that mostly deal with chronic, and untreated, psychotic patients.

Chiliza et al.^31^ examined the feasibility and effectiveness of a depot antipsychotic combined with an assertive monitoring program (AMP) using exploratory noncomparative study methodology. Participants (*n* = 207) were assessed, from the baseline up to 1 year. There was a significant improvement in positive and negative symptom scores (*P* ≤ .001), as well as quality of life (*P* ≤ .001) and authors concluded that in environments with limited resources, combining a depot antipsychotic with assertive monitoring is feasible and may be an effective treatment for first-episode schizophrenia. Ventura et al.^30^ demonstrated the feasibility of developing a clinical high-risk program (CHiRP) to identify youth at clinical high risk (CHR). Patients (*n* = 10) exhibiting possible signs of CHR were assessed by the CHiRP assessment team and provided with psychosocial and pharmacological management. Follow-up assessments were done at baseline, 3 months, and 6 months. The findings indicated the feasibility of implementing the methods established in HIC within LMIC settings. The study was carried out in Tunisia and recognized by the Early Intervention in Mental Health Association as the first CHR program in Africa.

### The Adaptation of EIP in LMIC

A detailed description of the characteristics of the EIP services is given in table 4 (supplementary appendix 9). We mapped the components of EIP as described in the included studies against the 7 standards provided by NICE guidelines^16^ for assessing the adaptation of EIP in LMIC settings.

1. *Referral and Assessment*: Referrals to EIP services were done by the hospitals, general practitioners, families or caregivers, and young people themselves.^34^ The assessment was carried out by psychiatrists^34,35,38^ using DSM-IV^34,38^ and ICD-10 criteria.^35^ Assessments included the Positive and Negative Syndrome Scale and Social and Occupational Functioning Assessment Scale.^34^ Singh et al. found that faith healing was the predominant kind of health-seeking behavior in North India, and it was strongly correlated with the prolonged duration of FEP in South India. It was observed that the treatment gap could be bridged by a collaborative model between the faith healers and the current healthcare system, creating a referral pathway.^35^
2. *Care Coordinator*: Case managers play a crucial role in facilitating the coordination of healthcare services for individual patients, while also providing non-pharmacological interventions.^38^
3. *Pharmacotherapy*: The feasibility of protocol-based management of FEP in the selection of antipsychotic medication, mode of administration, and monitoring of side effects has been demonstrated.^34–36^ The protocol-based management was delivered by a multidisciplinary team consisting of psychiatrists and case managers working in the Specialist FEP. A significant proportion of individuals used first-generation antipsychotics (*n* = 129, 81.13%) in contrast to second-generation antipsychotics (SGAs) (*n* = 3, 1.89%).^36^
4. *Psychoeducation and Support for Carers and Families:* Individual^36^ and family^34–36^ psychoeducation was delivered by a clinical psychologist. It was reported that the involvement of family members in treatment is a strong predictor of service engagement as the dropout rate observed at the Indian site (5.4%) was considerably lower than at the Canadian site (18.95%).^34^ Mottaghipour et al.^37^ examined the effectiveness of training health professionals to conduct family psychoeducation. The sessions were recorded and analyzed with scoring based on the manual’s content. Multiple family group education sessions in a hospital setting had a higher rate (79%) compared to the single-family home-based psychoeducation sessions (69%).
5. *Supported Employment Programs and Vocational Rehabilitation:* Using a retrospective chart review, Mwesiga et al.^36^ evaluated the quality of the individual and group-level interventions provided to FEP patients in Uganda. They reported limited provision of assistance to patients in terms of employment and vocational plans (*n* = 4, 2.5%) that were offered to only 1 and 4 patients, respectively, in a sample of 156 patients.
6. *Physical Health Interventions and Monitoring:* In their study, Mwesiga et al.^36^ assessed the monitoring of medication side effects and metabolic changes such as Body Mass Index (BMI), cholesterol, Random Blood Sugar (RBS), and weight gain, and reported that a significant proportion of participants exhibited poor monitoring of antipsychotic medication side effects (*n* = 39, 25%) and metabolic changes (*n* = 4, 2.5%).
7. *Evaluation and Quality Improvement*: Mwesiga et al.^36^ used the FEP Services Fidelity Scale to assess the quality of the services provided for each component of the specialized service. Iyer et al.^39^ developed “show me you care”: a new measure to explicitly evaluate the conduct and treatment approach of the treating team. Singh et al. found that faith healing was the predominant kind of health-seeking behavior in North India, and it was strongly correlated with the prolonged duration of FEP in South India.

### Quality Assessment

The quality criteria of the included study were assessed using the appropriate checklist for different study designs.^19^ The details of the quality assessment are provided in supplementary appendix 6. Out of the 4 RCTs, 1 had a low risk of bias, 2 had a high risk of bias, and 1 trial was classified as having some concerns. The quasi-experimental study was categorized as having a high risk of bias. Seven prospective studies were assessed using the STROBE checklist for cohort studies.^20^ Three studies failed to include key elements of study design. Only 2 studies mentioned how the sample size was arrived at. Five observational cross-sectional studies were included in the assessment. Four studies failed to mention proper sample size calculation and it was unclear whether study participants were sampled appropriately. Overall appraisal of the qualitative study was to be included in the review.

## Discussion

We used a comprehensive search strategy to map all evidence and found 18 studies from 6 out of 82 LMIC, highlighting the paucity of evidence. Around 90% of children and adolescents worldwide live in an LMIC, where they form half of the population.^40^ Considering that the peak onset of FEP is between 13 and 24 years,^41^ almost 90% of future FEP incidence would lie in these countries.^42^ Lack of effective interventions during the critical initial period of 2–5 years after the onset of psychosis would potentially condemn a substantial proportion of these young persons with disabling and often life-threatening chronic psychosis over the long-term follow-up.^11,12^

Despite the limited evidence in a few countries, we found that (a) The essential components of EIP can be adapted and provided in resource-poor settings and it may be feasible to establish these services in LMIC. (b) The adaptations will need to consider the high consultation rates with traditional healers, involving family and modifications in the role of different team members in EIP. (c) The effectiveness and metabolic side effects of SGAs are probably similar in the FEP population in these countries, and interventions to counteract the metabolic side effects are nonexistent or limited. (d) Mobile technology interventions such as SMS reminders can improve treatment engagement in this population. (e) The group interventions can help to reduce EE among the carers of FEP. We also aimed to examine the cost-effectiveness of EIS in LMIC but none of the included studies provided a cost-effective evaluation.

One previous systematic review^43^ examined the care pathways for psychosis in LMIC and highlighted the role of traditional healers in the care pathways. Despite the evidence that TSHs play a crucial role in treating FEP in LMIC, long periods of treatment by these healers significantly contribute to the exceptionally long DUP The role of TSH in schizophrenia care in LMIC is well known^14,22^ but the literature on how traditional healers can be involved in the early identification and management of FEP is sparse. A model of EIP in LMIC will need training and involvement of TSH in the early detection and management of FEP. A recent study^22^ has proposed a model for involving TSH in the early detection and management of FEP and evaluating its feasibility but this has not been evaluated fully.

Our review showed that the 7 key components of EIP services identified in NICE^16^ recommendations could be adapted for use in LMIC. The major modifications for implementing EIP include the following: (a) A collaborative care pathway between the faith healers and the healthcare professionals^35^ and referrals from a wide range of care providers ranging from general hospitals to young people themselves.^34^ (b) The adaptation of pharmacological management in view of limited availability of SGAs and use of long-acting depot antipsychotic.^28,36^ (c) Greater role of the family in care provision and in maintaining treatment engagement.^34,37^ (d) Case manager role adopted by a clinician in view of a limited number of trained professionals. It was clear that standalone EIP teams are not feasible in LMIC, and the service will be provided by existing mental health teams.

There are certain elements of EIS that can be adapted relatively easily in LMIC. This would include, eg, providing evidence-based pharmacological treatment for managing FEP, as almost all SGAs are available in these countries. Other interventions such as providing cognitive therapy will need cultural adaptation and using innovative approaches to deliver therapy in view of very low number of CBT therapists available in these countries. The organization of services will also be different, as EIS in these countries can be stand-alone services as in HIC. Finally, EIS services in LMIC will have certain unique features that do not fit well within the 7 key elements of good EIS as described in NICE guidance. This includes cultural and religious aspects of care such as including TSH in the care pathways for EIS, These aspects of adaptation of EIS in LMIC need to be identified and evaluated in future research studies.

The studies involving the use of antipsychotics highlighted the increased rates of metabolic side effects in the young FEP population.^44^ Patients with schizophrenia die about 10 years earlier than the general population due to cardiovascular disease.^44^ The mortality and morbidity may be even greater in South Asian populations who are at higher risk of developing cardiovascular disease with poorer outcomes^45^ and limited health facilities. This requires careful adaptation of current guidelines for LMIC in selecting and monitoring the use of SGAs.

Our literature review identified a few important gaps in providing EIP for LMIC. There was little evidence of the involvement of primary care in EIP, reflecting the lack of involvement of general practice in the care of psychosis in LMIC. Similarly, task-sharing and task-shifting approaches^46^ which have been widely used for the scale-up and increasing the accessibility of mental health services in LMIC were rarely employed in adapting the EIP in the included studies. We also identified limited provision of educational and vocational support. Only 1 study reported assistance to patients in terms of employment and vocational plans and found only a few patients receiving this in a cohort of FEP patients. Adequate employment and educational support for young people with FEP is crucial in developing, recovering, and maintaining their occupational skills.

Reducing the unacceptably long DUP in LMIC must be a key target of EIP services and hence there is a need for studies to detect and manage young persons during the prodrome. Interventions for the long DUP require phase-specific medical and psychological treatments in EIS and reducing delays in treatment following the onset of psychosis through early recognition.^47^ However, we could find only 1 study that described the potential development of a CHiRP intended to identify young individuals at CHR. This represents an important gap in evidence and clinical practice and needs to be addressed in future research.

Limitations of this study include a high risk of bias in most of the included studies. Key concepts such as FEP were not clearly defined in several studies and there was no controlled evaluation of EIP. It is difficult to generalize the findings as the studies were published by only 6 LMIC and were almost entirely done in specialized settings. The LMIC are not a homogenous group, these countries have diverse populations, cultures, and socioeconomic conditions and hence the findings cannot be generalized across all LMIC. We did include gray literature in the search strategy and it is possible that studies describing health services or programs may have been missed. The cost-effectiveness of EIP interventions or services has not been studied in LMIC settings. However, evidence from HIC suggests that EIP can have an incremental cost-effectiveness ratio of approximately $51 600 per Quality Adjusted Life Year.

From a societal perspective, this translates into much fewer hospitalizations and more years of employment throughout the remaining life expectancy of young FEP patients.^48^ In LMIC, the impacts would be much greater given the existing huge treatment gaps. It is therefore crucial to develop and evaluate EIP services in LMIC. This review provides a framework for the development of a service model for EIP in resource-poor settings and interventions that can be used to treat people with FEP. While specialist EIP services will not be feasible in these countries, it is possible to provide these services based on a public health approach.^12^

Future research needs to focus on controlled evaluation of EIP, implement and evaluate the evidence-based guidelines for managing the FEP in LMIC settings, and agree on quality standards that can be used for monitoring the quality of services. Future research needs to consider implementation frameworks such as RE-AIM (Reach, Effectiveness, Adoption, Implementation, Maintenance) that help to evaluate the implementation as well as the outcome of the service. The studies included in this review paid little attention to the patient and public involvement and it will be important to include the service users in all stages of research in this area.

## Conclusion

The available evidence suggests that EIP can be delivered in LMIC by adapting cultural factors and considering resource constraints. These adaptations might include partnering with traditional healers, engaging nonspecialist healthcare personnel, leveraging mobile technology, optimizing the utilization of long-acting antipsychotic medications, and closely monitoring the potential side effects of antipsychotics. Further research is needed using the implementation research methodologies to evaluate the effectiveness and cost-effectiveness of EIP in LMIC.

## Supplementary Material

sbae025_suppl_Supplementary_Appendix_1

sbae025_suppl_Supplementary_Appendix_2

sbae025_suppl_Supplementary_Appendix_3

sbae025_suppl_Supplementary_Appendix_4

sbae025_suppl_Supplementary_Appendix_5

sbae025_suppl_Supplementary_Appendix_6

sbae025_suppl_Supplementary_Appendix_7

sbae025_suppl_Supplementary_Appendix_8

sbae025_suppl_Supplementary_Appendix_9

## Data Availability

According to UK research councils’ Common Principles on Data Policy, we are working toward making data supporting this study available and access to extracted data will be available upon reasonable written request.
