# Supplementary material for: Early Intervention in Psychosis and Management of First Episode Psychosis in Low- and Lower-Middle-Income Countries: A Systematic Review
Source: Schizophr Bull. 2024 Mar 25;50(3):521–32. doi: 10.1093/schbul/sbae025 (PMC11059814; doi:10.1093/schbul/sbae025)
Supplement: sbae025_suppl_Supplementary_Appendix_2 [file sbae025_suppl_supplementary_appendix_2.docx]

**Appendix 2**

**Eligibility criteria outlined using PICO.**

| Participants/Population | - Studies done on individuals with the diagnosis of FEP. - Studies describing services for people in their early stages of psychosis, including FEP and Clinical High-Risk of Psychosis (CHR-P) regardless of age, gender, location, or comorbidities |
| --- | --- |
| Intervention | - Both pharmacological and non-pharmacological interventions (psychological, social, psychosocial) - Psychosis-focused therapy such as cognitive behavioural therapy, relaxation exercises, debriefing, pharmacotherapy, occupational therapy, and psychoeducation. |
| Comparator/Control | - Placebo for pharmacological interventions and treatment as usual (TAU) for psychological/social interventions. - Comparison group engaging in non-specific psychosocial activity (i.e., Attention control). |
| Outcome | - Possible treatment options, such as pharmacological, psychological, or psychosocial interventions may have the potential to reduce psychotic symptoms. - Components of EIP interventions/services - Evidence of effectiveness and cost-effectiveness - Implementation strategies |
| Setting | - Restricted to LMIC |
| Type of studies | - Randomized controlled trials, non-randomized controlled trials, qualitative studies, observational, analytical studies, and quantitative study designs. |
| Publication date | - Studies published from 1980 onwards |
| Language | - Available in English only |
| Key: First Episode Psychosis (FEP), Clinical High-Risk of Psychosis (CHR-P) Low and Lower-Middle-Income-Countries (LMIC) Early Intervention in Psychosis (EIP) | |
