# Supplementary material for: Early Intervention in Psychosis and Management of First Episode Psychosis in Low- and Lower-Middle-Income Countries: A Systematic Review
Source: Schizophr Bull. 2024 Mar 25;50(3):521–32. doi: 10.1093/schbul/sbae025 (PMC11059814; doi:10.1093/schbul/sbae025)
Supplement: sbae025_suppl_Supplementary_Appendix_3 [file sbae025_suppl_supplementary_appendix_3.docx]

|  | **Author** | **Definition of FEP** |
| --- | --- | --- |
| 1 | Rangaswamy et al., 2012; Malla et al., 2020,  , Iyer et al., 2022  ,Iyer et al., 2010  Vaitheswaran et al., 2021, lyer et al.,2023) | FEP must have a diagnosis of affective psychosis or schizophrenia spectrum psychotic disorder in DSM-IV (American Psychiatric Association, 2000) and must not have taken antipsychotic medication for more than 30 days since the onset of psychosis. |
| 2 | Sadath et al., 2017 | Patients diagnosed by a consultant psychiatrist with any non-affective psychosis of less than 5 years of duration. |
| 3 | Thomas et al., 2017 | Persons who present at a clinical setting who have never previously sought care for psychosis at an appropriate clinical setting where a certified psychiatrist makes a diagnosis. |
| 4 | Saddichha et al., 2008 | Patients who have not previously been exposed to drugs and have been diagnosed with first-episode schizophrenia according to the DSM-IV. |
| 5 | Modabbernia et al., 2014 | Diagnosis of schizophrenia based on DSM-IV. The patients are required to be in their first episode. (Patients with a history of taking olanzapine in the past three months were excluded) |
| 6 | Kaur et al., 2023 | Individuals diagnosed with schizophrenia according to the (DSM-5) in the first episode patients of schizophrenia meeting the DSM-fifth edition (DSM-5). Drug-naive or not using any psychiatric medication for more than two weeks with a less than one-year illness duration. |
| 7 | Tabatabaee et al., 2008 | The presence of psychotic symptoms, including delusions, hallucinations, disorganized speech, and/or substantially disorganized or catatonic behaviours, along with the current episode representing the first episode of a major psychiatric disorder. |
| 9 | Chiliza et al., 2016 | Patients meeting DSM-IV criteria. Participants who had been exposed to antipsychotic medication for a duration exceeding four weeks were excluded from the study |
| 10 | Singh et al., 2023 | First presentation with psychotic symptoms or less than 30 days of treatment with antipsychotics in any previous treatment episode and have one of the ICD-10 diagnoses. |
| 11 | Adhikari, 2014, Mwesiga et al.,2021, Mottaghipour et al.,2010 | FEP definitions not given properly |
| 12 | Ventura et al.,2021 | Research article is about clinical high-risk program for psychosis |
| **FEP**- First Episode Psychosis, **DSM-IV**- Diagnostic and Statistical Manual of Mental Disorders-Fourth Edition, **ICD**-International Classification of diseases | | |

**Appendix 3**

**First Episode Psychosis definitions of the included studies in the review.**
