# Supplementary material for: Early Intervention in Psychosis and Management of First Episode Psychosis in Low- and Lower-Middle-Income Countries: A Systematic Review
Source: Schizophr Bull. 2024 Mar 25;50(3):521–32. doi: 10.1093/schbul/sbae025 (PMC11059814; doi:10.1093/schbul/sbae025)
Supplement: sbae025_suppl_Supplementary_Appendix_4 [file sbae025_suppl_supplementary_appendix_4.docx]

**Appendix 4**

**Search strategy**

**The base structure of the search**

The databases/platforms used were OVID Medline, OVID Embase, Ebsco CINAHL & Proquest PsycINFO. All databases restricted searches to Title (TI) OR Abstract (AB).

**Templates for terms adapted to specific databases**

1. **LMIC country names**

afghanistan OR albania OR algeria OR american samoa OR angola OR (antigua and Barbuda) OR antigua OR barbuda OR argentina OR armenia OR armenian OR aruba OR azerbaijan OR bahrain OR bangladesh OR barbados OR republic of belarus OR belarus OR byelarus OR belorussia OR byelorussian OR belize OR british honduras OR benin OR dahomey OR bhutan OR bolivia OR (bosnia and Herzegovina) OR bosnia OR herzegovina OR botswana OR bechuanaland OR brazil OR brasil OR bulgaria OR burkina faso OR burkina fasso OR upper volta OR burundi OR urundi OR cabo verde OR cape verde OR cambodia OR kampuchea OR khmer republic OR cameroon OR cameron OR cameroun OR central african republic OR ubangi shari OR chad OR chile OR china OR colombia OR comoros OR comoro islands OR iles comores OR mayotte OR democratic republic of the congo OR democratic republic congo OR congo OR zaire OR costa rica OR cote divoire OR cote d ivoire OR ivory coast OR croatia OR cuba OR cyprus OR czech republic OR czechoslovakia OR djibouti OR french somaliland OR dominica OR dominican republic OR ecuador OR egypt OR united arab republic OR el salvador OR equatorial guinea OR spanish guinea OR eritrea OR estonia OR eswatini OR swaziland OR ethiopia OR fiji OR gabon OR gabonese republic OR gambia OR GEORGIA OR georgian OR ghana OR gold coast OR gibraltar OR greece OR grenada OR guam OR guatemala OR guinea OR guinea bissau OR guyana OR british guiana OR haiti OR hispaniola OR honduras OR hungary OR india OR indonesia OR timor OR iran OR iraq OR isle of man OR jamaica OR jordan OR kazakhstan OR kazakh OR kenya OR democratic peoples republic of korea OR republic of korea OR north korea OR south korea OR korea OR kosovo OR kyrgyzstan OR kirghizia OR kirgizstan OR kyrgyz republic OR kirghiz OR laos OR lao pdr OR lao peoples democratic republic OR latvia OR lebanon OR lebanese republic OR lesotho OR basutoland OR liberia OR libya OR libyan arab jamahiriya OR lithuania OR macau OR macao OR republic of north macedonia OR macedonia OR madagascar OR malagasy republic OR malawi OR nyasaland OR malaysia OR malay federation OR malaya federation OR maldives OR indian ocean islands OR indian ocean OR mali OR malta OR micronesia OR federated states of micronesia OR kiribati OR marshall islands OR nauru OR northern mariana islands OR palau OR tuvalu OR mauritania OR mauritius OR mexico OR moldova OR moldovian OR mongolia OR montenegro OR morocco OR ifni OR mozambique OR portuguese east africa OR myanmar OR burma OR namibia OR nepal OR netherlands antilles OR nicaragua OR niger OR nigeria OR oman OR muscat OR pakistan OR panama OR papua new guinea OR new guinea OR paraguay OR peru OR philippines OR philipines OR phillipines OR phillippines OR poland OR polish peoples republic OR portugal OR portuguese republic OR puerto rico OR romania OR russia OR russian federation OR ussr OR soviet union OR union of soviet socialist republics OR rwanda OR ruanda OR samoa OR pacific islands OR polynesia OR samoan islands OR navigator island OR navigator islands OR (sao tome and principe) OR saudi arabia OR senegal OR serbia OR seychelles OR sierra leone OR slovakia OR slovak republic OR slovenia OR melanesia OR solomon island OR solomon islands OR norfolk island OR norfolk islands OR somalia OR south africa OR south sudan OR sri lanka OR ceylon OR (saint kitts and nevis) OR (st. kitts and nevis) OR saint lucia OR (st. lucia) OR (saint vincent and the grenadines) OR saint vincent OR (st. Vincent) OR grenadines OR sudan OR suriname OR surinam OR dutch guiana OR netherlands guiana OR syria OR syrian arab republic OR tajikistan OR tadjikistan OR tadzhikistan OR tadzhik OR tanzania OR tanganyika OR thailand OR siam OR timor leste OR east timor OR togo OR togolese republic OR tonga OR (trinidad and Tobago) OR trinidad OR tobago OR tunisia OR turkey OR turkmenistan OR turkmen OR uganda OR ukraine OR uruguay OR uzbekistan OR uzbek OR vanuatu OR new hebrides OR venezuela OR vietnam OR viet nam OR middle east OR west bank OR gaza OR palestine OR yemen OR yugoslavia OR zambia OR zimbabwe OR northern rhodesia OR global south OR africa south of the sahara OR sub-saharan africa OR subsaharan africa OR africa, central OR central africa OR africa, northern OR north africa OR northern africa OR magreb OR maghrib OR sahara OR africa, southern OR southern africa OR africa, eastern OR east africa OR eastern africa OR africa, western OR west africa OR western africa OR west indies OR indian ocean islands OR caribbean OR central america OR latin america OR (south and central America) OR south america OR asia, central OR central asia OR asia, northern OR north asia OR northern asia OR asia, southeastern OR southeastern asia OR south eastern asia OR southeast asia OR south east asia OR asia, western OR western asia OR europe, eastern OR east europe OR eastern Europe OR TAIWAN

**LMIC GENERIC TERMS**

LMIC OR LMICS OR (lower and middle income countries) OR LAMI countr* OR (developing ADJ2 (countr* OR econom* OR nation* OR world)) OR (less developed ADJ2 (countr* OR econom* OR nation* OR world)) OR (underdeveloped ADJ2 (countr* OR econom* OR nation* OR world)) OR (under developed ADJ2 (countr* OR econom* OR nation* OR world)) OR (middle income ADJ2 (countr* OR econom* OR nation* OR world)) OR (middle-income ADJ2 (countr* OR econom* OR nation* OR world)) OR (lower income ADJ2 (countr* OR econom* OR nation* OR world)) OR (low income ADJ2 (countr* OR econom* OR nation* OR world)) OR (lower-income ADJ2 (countr* OR econom* OR nation* OR world)) OR (low-income ADJ2 (countr* OR econom* OR nation* OR world)) OR (deprived ADJ2 (countr* OR econom* OR nation* OR world)) OR (poorer ADJ2 (countr* OR econom* OR nation* OR world)) OR (third world ADJ2 (countr* OR econom* OR nation* OR world)) OR emerging countr* OR emerging econom* OR transitional countr* OR transitional econom*

1. **First-episode psychosis/early intervention terms**

Early intervention in psychosis OR EIP OR first episode psychosis OR early intervention ADJ4 psychosis OR early intervention ADJ4 schizophrenia OR first episode intervention OR FEP OR ultra-high risk mental state OR at-risk mental state OR prodrome OR clinical high-risk ADJ2 psychosis OR care pathway* ADJ3 psychosis OR care pathway* ADJ3 schizophrenia

Subject heading appropriate to the database combined with AND (early intervention or first episode or ultra-high risk or at-risk)

**Ovid MEDLINE**

1 exp "schizophrenia spectrum and other psychotic disorders"/ or psychotic disorders/

2 (early intervention or first episode or ultra-high risk or at-risk).mp. [mp=title, book title, abstract, original title, name of substance word, subject heading word, floating sub-heading word, keyword heading word, organism supplementary concept word, protocol supplementary concept word, rare disease supplementary concept word, unique identifier, synonyms]

3 1 and 2

4 (Early intervention in psychosis or EIP or first-episode psychosis or (early intervention adj4 psychosis) or (early intervention adj4 schizophrenia) or first episode intervention or FEP or ultra-high risk mental state or at-risk mental state or prodrome or (clinical high-risk adj2 psychosis) or (care pathway* adj3 psychosis) or (care pathway* adj3 schizophrenia)).ti.ab.kf.hw.

7 (LMIC or LMICS or (lower and middle income countries) or LAMI countr* or (developing adj2 (countr* or econom* or nation* or world)) or (less developed adj2 (countr* or econom* or nation* or world)) or (underdeveloped adj2 (countr* or econom* or nation* or world)) or (under developed adj2 (countr* or econom* or nation* or world)) or (middle income adj2 (countr* or econom* or nation* or world)) or (middle-income adj2 (countr* or econom* or nation* or world)) or (lower income adj2 (countr* or econom* or nation* or world)) or (low income adj2 (countr* or econom* or nation* or world)) or (lower-income adj2 (countr* or econom* or nation* or world)) or (low-income adj2 (countr* or econom* or nation* or world)) or (deprived adj2 (countr* or econom* or nation* or world)) or (poorer adj2 (countr* or econom* or nation* or world)) or (third world adj2 (countr* or econom* or nation* or world)) or emerging countr* or emerging econom* or transitional countr* or transitional econom*).ti.ab.kf.hw.

8 (afghanistan or albania or algeria or american samoa or angola or (antigua and Barbuda) or antigua or barbuda or argentina or armenia or armenian or aruba or azerbaijan or bahrain or bangladesh or barbados or republic of belarus or belarus or byelarus or belorussia or byelorussian or belize or british honduras or benin or dahomey or bhutan or bolivia or (bosnia and Herzegovina) or bosnia or herzegovina or botswana or bechuanaland or brazil or brasil or bulgaria or burkina faso or burkina fasso or upper volta or burundi or urundi or cabo verde or cape verde or cambodia or kampuchea or khmer republic or cameroon or cameron or cameroun or central african republic or ubangi shari or chad or chile or china or colombia or comoros or comoro islands or iles comores or mayotte or democratic republic of the congo or democratic republic congo or congo or zaire or costa rica or cote divoire or cote d ivoire or ivory coast or croatia or cuba or cyprus or czech republic or czechoslovakia or djibouti or french somaliland or dominica or dominican republic or ecuador or egypt or united arab republic or el salvador or equatorial guinea or spanish guinea or eritrea or estonia or eswatini or swaziland or ethiopia or fiji or gabon or gabonese republic or gambia or GEORGIA or georgian or ghana or gold coast or gibraltar or greece or grenada or guam or guatemala or guinea or guinea bissau or guyana or british guiana or haiti or hispaniola or honduras or hungary or india or indonesia or timor or iran or iraq or isle of man or jamaica or jordan or kazakhstan or kazakh or kenya or democratic peoples republic of korea or republic of korea or north korea or south korea or korea or kosovo or kyrgyzstan or kirghizia or kirgizstan or kyrgyz republic or kirghiz or laos or lao pdr or lao peoples democratic republic or latvia or lebanon or lebanese republic or lesotho or basutoland or liberia or libya or libyan arab jamahiriya or lithuania or macau or macao or republic of north macedonia or macedonia or madagascar or malagasy republic or malawi or nyasaland or malaysia or malay federation or malaya federation or maldives or indian ocean islands or indian ocean or mali or malta or micronesia or federated states of micronesia or kiribati or marshall islands or nauru or northern mariana islands or palau or tuvalu or mauritania or mauritius or mexico or moldova or moldovian or mongolia or montenegro or morocco or ifni or mozambique or portuguese east africa or myanmar or burma or namibia or nepal or netherlands antilles or nicaragua or niger or nigeria or oman or muscat or pakistan or panama or papua new guinea or new guinea or paraguay or peru or philippines or philipines or phillipines or phillippines or poland or polish peoples republic or portugal or portuguese republic or puerto rico or romania or russia or russian federation or ussr or soviet union or union of soviet socialist republics or rwanda or ruanda or samoa or pacific islands or polynesia or samoan islands or navigator island or navigator islands or (sao tome and principe) or saudi arabia or senegal or serbia or seychelles or sierra leone or slovakia or slovak republic or slovenia or melanesia or solomon island or solomon islands or norfolk island or norfolk islands or somalia or south africa or south sudan or sri lanka or ceylon or (saint kitts and nevis) or (st kitts and nevis) or saint lucia or st lucia or (saint vincent and the grenadines) or saint vincent or st Vincent or grenadines or sudan or suriname or surinam or dutch guiana or netherlands guiana or syria or syrian arab republic or tajikistan or tadjikistan or tadzhikistan or tadzhik or tanzania or tanganyika or thailand or siam or timor leste or east timor or togo or togolese republic or tonga or (trinidad and Tobago) or trinidad or tobago or tunisia or turkey or turkmenistan or turkmen or uganda or ukraine or uruguay or uzbekistan or uzbek or vanuatu or new hebrides or venezuela or vietnam or viet nam or middle east or west bank or gaza or palestine or yemen or yugoslavia or zambia or zimbabwe or northern rhodesia or global south or africa south of the sahara or sub-saharan africa or subsaharan africa or africa, central or central africa or africa, northern or north africa or northern africa or magreb or maghrib or sahara or africa, southern or southern africa or africa, eastern or east africa or eastern africa or africa, western or west africa or western africa or west indies or indian ocean islands or caribbean or central america or latin america or (south and central America) or south america or asia, central or central asia or asia, northern or north asia or northern asia or asia, southeastern or southeastern asia or south eastern asia or southeast asia or south east asia or asia, western or western asia or europe, eastern or east europe or eastern Europe or TAIWAN).ti.ab.kf.hw.

9 7 or 8

10 6 and 9

11 limit 10 to (English language and yr="1980 -Current")

**OVID Embase**

1 exp psychosis/

2 (early intervention or first episode or ultra-high risk or at-risk).mp. [mp=title, abstract, heading word, drug trade name, original title, device manufacturer, drug manufacturer, device trade name, keyword heading word, floating subheading word, candidate term word]

3 1 and 2

4 (Early intervention in psychosis or EIP or first-episode psychosis or (early intervention adj4 psychosis) or (early intervention adj4 schizophrenia) or first episode intervention or FEP or ultra-high risk mental state or at-risk mental state or prodrome or (clinical high-risk adj2 psychosis) or (care pathway* adj3 psychosis) or (care pathway* adj3 schizophrenia)).mp. [mp=title, abstract, heading word, drug trade name, original title, device manufacturer, drug manufacturer, device trade name, keyword heading word, floating subheading word, candidate term word]

5 3 or 4

6 (LMIC or LMICS or (lower and middle income countries) or LAMI countr* or (developing adj2 (countr* or econom* or nation* or world)) or (less developed adj2 (countr* or econom* or nation* or world)) or (underdeveloped adj2 (countr* or econom* or nation* or world)) or (under developed adj2 (countr* or econom* or nation* or world)) or (middle income adj2 (countr* or econom* or nation* or world)) or (middle-income adj2 (countr* or econom* or nation* or world)) or (lower income adj2 (countr* or econom* or nation* or world)) or (low income adj2 (countr* or econom* or nation* or world)) or (lower-income adj2 (countr* or econom* or nation* or world)) or (low-income adj2 (countr* or econom* or nation* or world)) or (deprived adj2 (countr* or econom* or nation* or world)) or (poorer adj2 (countr* or econom* or nation* or world)) or (third world adj2 (countr* or econom* or nation* or world)) or emerging countr* or emerging econom* or transitional countr* or transitional econom*).mp. [mp=title, abstract, heading word, drug trade name, original title, device manufacturer, drug manufacturer, device trade name, keyword heading word, floating subheading word, candidate term word]

7 (afghanistan or albania or algeria or american samoa or angola or (antigua and Barbuda) or antigua or barbuda or argentina or armenia or armenian or aruba or azerbaijan or bahrain or bangladesh or barbados or republic of belarus or belarus or byelarus or belorussia or byelorussian or belize or british honduras or benin or dahomey or bhutan or bolivia or (bosnia and Herzegovina) or bosnia or herzegovina or botswana or bechuanaland or brazil or brasil or bulgaria or burkina faso or burkina fasso or upper volta or burundi or urundi or cabo verde or cape verde or cambodia or kampuchea or khmer republic or cameroon or cameron or cameroun or central african republic or ubangi shari or chad or chile or china or colombia or comoros or comoro islands or iles comores or mayotte or democratic republic of the congo or democratic republic congo or congo or zaire or costa rica or cote divoire or cote d ivoire or ivory coast or croatia or cuba or cyprus or czech republic or czechoslovakia or djibouti or french somaliland or dominica or dominican republic or ecuador or egypt or united arab republic or el salvador or equatorial guinea or spanish guinea or eritrea or estonia or eswatini or swaziland or ethiopia or fiji or gabon or gabonese republic or gambia or GEORGIA or georgian or ghana or gold coast or gibraltar or greece or grenada or guam or guatemala or guinea or guinea bissau or guyana or british guiana or haiti or hispaniola or honduras or hungary or india or indonesia or timor or iran or iraq or isle of man or jamaica or jordan or kazakhstan or kazakh or kenya or democratic peoples republic of korea or republic of korea or north korea or south korea or korea or kosovo or kyrgyzstan or kirghizia or kirgizstan or kyrgyz republic or kirghiz or laos or lao pdr or lao peoples democratic republic or latvia or lebanon or lebanese republic or lesotho or basutoland or liberia or libya or libyan arab jamahiriya or lithuania or macau or macao or republic of north macedonia or macedonia or madagascar or malagasy republic or malawi or nyasaland or malaysia or malay federation or malaya federation or maldives or indian ocean islands or indian ocean or mali or malta or micronesia or federated states of micronesia or kiribati or marshall islands or nauru or northern mariana islands or palau or tuvalu or mauritania or mauritius or mexico or moldova or moldovian or mongolia or montenegro or morocco or ifni or mozambique or portuguese east africa or myanmar or burma or namibia or nepal or netherlands antilles or nicaragua or niger or nigeria or oman or muscat or pakistan or panama or papua new guinea or new guinea or paraguay or peru or philippines or philipines or phillipines or phillippines or poland or polish peoples republic or portugal or portuguese republic or puerto rico or romania or russia or russian federation or ussr or soviet union or union of soviet socialist republics or rwanda or ruanda or samoa or pacific islands or polynesia or samoan islands or navigator island or navigator islands or (sao tome and principe) or saudi arabia or senegal or serbia or seychelles or sierra leone or slovakia or slovak republic or slovenia or melanesia or solomon island or solomon islands or norfolk island or norfolk islands or somalia or south africa or south sudan or sri lanka or ceylon or (saint kitts and nevis) or (st kitts and nevis) or saint lucia or st lucia or (saint vincent and the grenadines) or saint vincent or st Vincent or grenadines or sudan or suriname or surinam or dutch guiana or netherlands guiana or syria or syrian arab republic or tajikistan or tadjikistan or tadzhikistan or tadzhik or tanzania or tanganyika or thailand or siam or timor leste or east timor or togo or togolese republic or tonga or (trinidad and Tobago) or trinidad or tobago or tunisia or turkey or turkmenistan or turkmen or uganda or ukraine or uruguay or uzbekistan or uzbek or vanuatu or new hebrides or venezuela or vietnam or viet nam or middle east or west bank or gaza or palestine or yemen or yugoslavia or zambia or zimbabwe or northern rhodesia or global south or africa south of the sahara or sub-saharan africa or subsaharan africa or africa, central or central africa or africa, northern or north africa or northern africa or magreb or maghrib or sahara or africa, southern or southern africa or africa, eastern or east africa or eastern africa or africa, western or west africa or western africa or west indies or indian ocean islands or caribbean or central america or latin america or (south and central America) or south america or asia, central or central asia or asia, northern or north asia or northern asia or asia, southeastern or southeastern asia or south eastern asia or southeast asia or south east asia or asia, western or western asia or europe, eastern or east europe or eastern Europe or TAIWAN).mp. [mp=title, abstract, heading word, drug trade name, original title, device manufacturer, drug manufacturer, device trade name, keyword heading word, floating subheading word, candidate term word]

8 6 or 7

9 5 and 8

10 limit 9 to (English language and yr="1980 -Current")

**EBSCO CINAHL**

1 LMIC Countries

afghanistan OR albania OR algeria OR american samoa OR angola OR (antigua and Barbuda) OR antigua OR barbuda OR argentina OR armenia OR armenian OR aruba OR azerbaijan OR bahrain OR bangladesh OR barbados OR republic of belarus OR belarus OR byelarus OR belorussia OR byelorussian OR belize OR british honduras OR benin OR dahomey OR bhutan OR bolivia OR (bosnia and Herzegovina) OR bosnia OR herzegovina OR botswana OR bechuanaland OR brazil OR brasil OR bulgaria OR burkina faso OR burkina fasso OR upper volta OR burundi OR urundi OR cabo verde OR cape verde OR cambodia OR kampuchea OR khmer republic OR cameroon OR cameron OR cameroun OR central african republic OR ubangi shari OR chad OR chile OR china OR colombia OR comoros OR comoro islands OR iles comores OR mayotte OR democratic republic of the congo OR democratic republic congo OR congo OR zaire OR costa rica OR cote divoire OR cote d ivoire OR ivory coast OR croatia OR cuba OR cyprus OR czech republic OR czechoslovakia OR djibouti OR french somaliland OR dominica OR dominican republic OR ecuador OR egypt OR united arab republic OR el salvador OR equatorial guinea OR spanish guinea OR eritrea OR estonia OR eswatini OR swaziland OR ethiopia OR fiji OR gabon OR gabonese republic OR gambia OR GEORGIA OR georgian OR ghana OR gold coast OR gibraltar OR greece OR grenada OR guam OR guatemala OR guinea OR guinea bissau OR guyana OR british guiana OR haiti OR hispaniola OR honduras OR hungary OR india OR indonesia OR timor OR iran OR iraq OR isle of man OR jamaica OR jordan OR kazakhstan OR kazakh OR kenya OR democratic peoples republic of korea OR republic of korea OR north korea OR south korea OR korea OR kosovo OR kyrgyzstan OR kirghizia OR kirgizstan OR kyrgyz republic OR kirghiz OR laos OR lao pdr OR lao peoples democratic republic OR latvia OR lebanon OR lebanese republic OR lesotho OR basutoland OR liberia OR libya OR libyan arab jamahiriya OR lithuania OR macau OR macao OR republic of north macedonia OR macedonia OR madagascar OR malagasy republic OR malawi OR nyasaland OR malaysia OR malay federation OR malaya federation OR maldives OR indian ocean islands OR indian ocean OR mali OR malta OR micronesia OR federated states of micronesia OR kiribati OR marshall islands OR nauru OR northern mariana islands OR palau OR tuvalu OR mauritania OR mauritius OR mexico OR moldova OR moldovian OR mongolia OR montenegro OR morocco OR ifni OR mozambique OR portuguese east africa OR myanmar OR burma OR namibia OR nepal OR netherlands antilles OR nicaragua OR niger OR nigeria OR oman OR muscat OR pakistan OR panama OR papua new guinea OR new guinea OR paraguay OR peru OR philippines OR philipines OR phillipines OR phillippines OR poland OR polish peoples republic OR portugal OR portuguese republic OR puerto rico OR romania OR russia OR russian federation OR ussr OR soviet union OR union of soviet socialist republics OR rwanda OR ruanda OR samoa OR pacific islands OR polynesia OR samoan islands OR navigator island OR navigator islands OR (sao tome and principe) OR saudi arabia OR senegal OR serbia OR seychelles OR sierra leone OR slovakia OR slovak republic OR slovenia OR melanesia OR solomon island OR solomon islands OR norfolk island OR norfolk islands OR somalia OR south africa OR south sudan OR sri lanka OR ceylon OR (saint kitts and nevis) OR (st. kitts and nevis) OR saint lucia OR (st. lucia) OR (saint vincent and the grenadines) OR saint vincent OR (st. Vincent) OR grenadines OR sudan OR suriname OR surinam OR dutch guiana OR netherlands guiana OR syria OR syrian arab republic OR tajikistan OR tadjikistan OR tadzhikistan OR tadzhik OR tanzania OR tanganyika OR thailand OR siam OR timor leste OR east timor OR togo OR togolese republic OR tonga OR (trinidad and Tobago) OR trinidad OR tobago OR tunisia OR turkey OR turkmenistan OR turkmen OR uganda OR ukraine OR uruguay OR uzbekistan OR uzbek OR vanuatu OR new hebrides OR venezuela OR vietnam OR viet nam OR middle east OR west bank OR gaza OR palestine OR yemen OR yugoslavia OR zambia OR zimbabwe OR northern rhodesia OR global south OR africa south of the sahara OR sub-saharan africa OR subsaharan africa OR africa, central OR central africa OR africa, northern OR north africa OR northern africa OR magreb OR maghrib OR sahara OR africa, southern OR southern africa OR africa, eastern OR east africa OR eastern africa OR africa, western OR west africa OR western africa OR west indies OR indian ocean islands OR caribbean OR central america OR latin america OR (south and central America) OR south america OR asia, central OR central asia OR asia, northern OR north asia OR northern asia OR asia, southeastern OR southeastern asia OR south eastern asia OR southeast asia OR south east asia OR asia, western OR western asia OR europe, eastern OR east europe OR eastern Europe OR TAIWAN

2 LMIC GENERIC TERMS

LMIC OR LMICS OR (lower and middle income countries) OR LAMI countr* OR (developing N1 (countr* OR econom* OR nation* OR world)) OR (less developed N1 (countr* OR econom* OR nation* OR world)) OR (underdeveloped N1 (countr* OR econom* OR nation* OR world)) OR (under developed N1 (countr* OR econom* OR nation* OR world)) OR (middle income N1 (countr* OR econom* OR nation* OR world)) OR (middle-income N1 (countr* OR econom* OR nation* OR world)) OR (lower income N1 (countr* OR econom* OR nation* OR world)) OR (low income N1 (countr* OR econom* OR nation* OR world)) OR (lower-income N1 (countr* OR econom* OR nation* OR world)) OR (low-income N1 (countr* OR econom* OR nation* OR world)) OR (deprived N1 (countr* OR econom* OR nation* OR world)) OR (poorer N1 (countr* OR econom* OR nation* OR world)) OR (third world N1 (countr* OR econom* OR nation* OR world)) OR emerging countr* OR emerging econom* OR transitional countr* OR transitional econom*

3 Combined Country OR generic term

4 First-episode psychosis/early intervention terms

Early intervention in psychosis OR EIP OR first episode psychosis OR early intervention W3 psychosis OR early intervention W3 schizophrenia OR first episode intervention OR FEP OR ultra-high risk mental state OR at-risk mental state OR prodrome OR clinical high risk W1 psychosis OR care pathway* N2 psychosis OR care pathway* N2 schizophrenia

5 (Combined country & generic term) AND first episode psychosis terms

6 (MH "Affective Disorders, Psychotic+") OR (MH "Schizophrenia+")

7 early intervention or first episode or ultra-high risk or at-risk (This search line run in Title field (TI), Abstract field (AB) & Word in Subject heading (MW) )

8 6 and 7 (Subject headings re psychosis/schizophrenia with first episode related terms)

9 3 and 8 (country AND (combined subject headings AND first episode)

10 5 OR 9

11 Limited to 1980-current, English language

**Proquest PsycINFO**

LMIC Country terms

afghanistan OR albania OR algeria OR “american samoa” OR angola OR “antigua and Barbuda” OR antigua OR barbuda OR argentina OR armenia OR armenian OR aruba OR azerbaijan OR bahrain OR bangladesh OR barbados OR “republic of belarus” OR belarus OR byelarus OR belorussia OR byelorussian OR belize OR “british honduras” OR benin OR dahomey OR bhutan OR bolivia OR “bosnia and Herzegovina” OR bosnia OR herzegovina OR botswana OR bechuanaland OR brazil OR brasil OR bulgaria OR burkina faso OR “burkina fasso” OR “upper volta” OR burundi OR urundi OR “cabo verde”OR “cape verde” OR cambodia OR kampuchea OR “khmer republic” OR cameroon OR cameron OR cameroun OR “central african republic” OR “ubangi shari” OR chad OR chile OR china OR colombia OR comoros OR “comoro islands” OR “iles comores” OR mayotte OR “democratic republic of the congo” OR “democratic republic congo” OR congo OR zaire OR “costa rica” OR “cote divoire” OR “cote d’ivoire” OR “ivory coast” OR croatia OR cuba OR cyprus OR “czech republic” OR czechoslovakia OR djibouti OR french somaliland OR dominica OR “dominican republic” OR ecuador OR egypt OR “united arab republic” OR “el Salvador” OR “equatorial guinea” OR “spanish guinea” OR eritrea OR estonia OR eswatini OR swaziland OR ethiopia OR fiji OR gabon OR “gabonese republic” OR gambia OR GEORGIA OR georgian OR ghana OR “gold coast” OR gibraltar OR greece OR grenada OR guam OR guatemala OR guinea OR “guinea Bissau” OR guyana OR “british Guiana” OR haiti OR hispaniola OR honduras OR hungary OR india OR indonesia OR timor OR iran OR iraq OR “isle of man” OR jamaica OR jordan OR kazakhstan OR kazakh OR kenya OR “democratic peoples republic of korea” OR “republic of korea” OR “north korea” OR “south korea” OR korea OR kosovo OR kyrgyzstan OR kirghizia OR kirgizstan OR “kyrgyz republic” OR kirghiz OR laos OR “lao pdr” OR “lao peoples democratic republic” OR latvia OR lebanon OR “lebanese republic” OR lesotho OR basutoland OR liberia OR libya OR “libyan arab Jamahiriya” OR lithuania OR macau OR macao OR “republic of north Macedonia” OR macedonia OR madagascar OR “malagasy republic” OR malawi OR nyasaland OR malaysia OR “malay federation” OR “malaya federation” OR maldives OR “indian ocean islands”OR “indian ocean” OR mali OR malta OR micronesia OR “federated states of Micronesia” OR kiribati OR “marshall islands” OR nauru OR “northern mariana islands” OR palau OR tuvalu OR mauritania OR mauritius OR mexico OR moldova OR moldovian OR mongolia OR montenegro OR morocco OR ifni OR mozambique OR “portuguese east Africa” OR myanmar OR burma OR namibia OR nepal OR “netherlands Antilles” OR antilles OR nicaragua OR niger OR nigeria OR oman OR muscat OR pakistan OR panama OR “papua new guinea” OR “new guinea” OR paraguay OR peru OR philippines OR philipines OR phillipines OR phillippines OR poland OR “polish peoples republic” OR portugal OR “portuguese republic” OR “puerto rico” OR romania OR russia OR “russian federation” OR ussr OR “soviet union” OR “union of soviet socialist republics” OR rwanda OR ruanda OR samoa OR “pacific islands” OR polynesia OR “samoan islands” OR “navigator island” OR “navigator islands” OR “sao tome and principe” OR “saudi arabia” OR senegal OR serbia OR seychelles OR “sierra leone” OR slovakia OR “slovak republic” OR slovenia OR melanesia OR “solomon island” OR “solomon islands” OR “norfolk island” OR “norfolk islands” OR somalia OR “South Africa” OR south sudan OR sri lanka OR ceylon OR “saint kitts and nevis” OR “st. kitts and nevis” OR “saint lucia” OR “st. lucia” OR “saint vincent and the grenadines” OR “saint Vincent” OR “st. Vincent” OR grenadines OR sudan OR suriname OR surinam OR “dutch Guiana” OR “netherlands Guiana” OR syria OR “syrian arab republic” OR tajikistan OR tadjikistan OR tadzhikistan OR tadzhik OR tanzania OR tanganyika OR thailand OR siam OR “timor leste” OR “east timor” OR togo OR “togolese republic” OR tonga OR “trinidad and Tobago” OR trinidad OR tobago OR tunisia OR turkey OR turkmenistan OR turkmen OR uganda OR ukraine OR uruguay OR uzbekistan OR uzbek OR vanuatu OR “new Hebrides” OR venezuela OR vietnam OR “viet nam” OR “middle east” OR “west bank” OR gaza OR palestine OR yemen OR yugoslavia OR zambia OR zimbabwe OR “northern Rhodesia” OR “global south” OR “africa south of the sahara” OR “sub-saharan Africa” OR “subsaharan Africa” OR “central africa” OR “north Africa” OR “northern Africa” OR magreb OR maghrib OR sahara OR “southern africa” OR “east Africa” OR “eastern Africa” OR “west africa“ OR “western africa“ OR “west indies” OR “indian ocean islands” OR caribbean OR “central America” OR “latin America” OR “south and central America” OR “south America” OR “central asia” OR “north asia” OR “northern asia” OR “southeastern asia” OR “south eastern asia” OR “southeast asia” OR “south east asia” OR “western asia” OR “east Europe” OR “eastern Europe” OR TAIWAN

1 Above search line run in Title & abstract field (TIAB) & Subject heading (SU)

LMIC GENERIC TERMS

LMIC OR LMICS OR “lower and middle income countries” OR LAMI countr* OR “developing N/2 (countr* OR econom* OR nation* OR world)” OR “less developed N/2 (countr* OR econom* OR nation* OR world)” OR “”underdeveloped N/2 (countr* OR econom* OR nation* OR world)” OR “under developed N/2 (countr* OR econom* OR nation* OR world)” OR “middle income N/2 (countr* OR econom* OR nation* OR world)” OR “middle-income N/2 (countr* OR econom* OR nation* OR world)” OR “lower income N/2 (countr* OR econom* OR nation* OR world)” OR “low income N/2 (countr* OR econom* OR nation* OR world)) OR “lower-income N/2 (countr* OR econom* OR nation* OR world)” OR (“low-income N/2 (countr* OR econom* OR nation* OR world)” OR “deprived N/2 (countr* OR econom* OR nation* OR world)” OR “poorer N/2 (countr* OR econom* OR nation* OR world)” OR “third world N/2 (countr* OR econom* OR nation* OR world)” OR “emerging countr*” OR “emerging econom*” OR “transitional countr*” OR “transitional econom*”

2 Above search line run in Title & abstract field (TIAB) & Subject heading (SU)

3 Combined Country OR generic term

First episode psychosis/early intervention terms

“Early intervention in psychosis” OR EIP OR “first episode psychosis” OR “early intervention N/3 psychosis” OR “early intervention N/3 schizophrenia” OR “first episode intervention” OR FEP OR “ultra-high risk mental state” OR “at-risk mental state” OR prodrome OR “clinical high risk N/2 psychosis” OR “care pathway*” N/2 psychosis” OR “care pathway* N/2 schizophrenia”

4 Above search line run in Title & abstract field (TIAB) & Subject heading (SU)

Update:

5 MAINSUBJECT.EXACT.EXPLODE("Psychosis") *(this includes schizophrenia)* AND (early intervention or first episode or ultra-high risk or at-risk) *(This search line run in Title & abstract field (TIAB) & Subject heading (SU))*

6 4 OR 5 (First episode psychosis OR Subject headings re psychosis/schizophrenia with first episode related terms)

7 3 and 6 country AND combined psychosis terms

10 Limited to 1980-2022, English Language

Update: Limited to 2022-23
