# Supplementary material for: Early Intervention in Psychosis and Management of First Episode Psychosis in Low- and Lower-Middle-Income Countries: A Systematic Review
Source: Schizophr Bull. 2024 Mar 25;50(3):521–32. doi: 10.1093/schbul/sbae025 (PMC11059814; doi:10.1093/schbul/sbae025)
Supplement: sbae025_suppl_Supplementary_Appendix_6 [file sbae025_suppl_supplementary_appendix_6.docx]

**Appendix 6**

**Quality Assessment**

1. **Randomized Controlled Trials- Revised Cochrane risk-of-bias tool for randomized trials (RoB 2)**

| Author | **Randomization process** | **Deviations from the intended interventions** | **Missing outcome data** | **Measurement of the outcome** | **Selection of the reported result** | **The overall risk of bias** |
| --- | --- | --- | --- | --- | --- | --- |
| Thomas et al., 2017 |  |  |  |  |  |  |
| Modabbernia et al., 2014 |  |  |  |  |  |  |
| Saddichha et al., 2008 |  |  |  |  |  |  |
| Kaur et al., 2023 |  |  |  |  |  |  |

Low risk Some concern High risk

1. **Non-randomized controlled trials- Risk Of Bias In Non-randomized Studies - of Exposure (ROBINS-E). Version 20 June 2023**

| Author | **Confounding** | **Measurement of the exposure** | **Selection of participants into the study (or into the analysis)** | **Post-exposure interventions** | **Missing data** | **Measurement of the outcome** | **Reported result** | **The overall risk of bias** |
| --- | --- | --- | --- | --- | --- | --- | --- | --- |
| Sadath et al., 2017 |  |  |  |  |  |  |  |  |

Low risk Some concern High risk

1. **Prospective studies- STROBE checklist for cohort studies**

| **Author** | **Study design** | **Study setting** | **Eligibility criteria** | **Variables** | **Data source** | **Bias** | **Study size** | **Quantitative variables** |
| --- | --- | --- | --- | --- | --- | --- | --- | --- |
| Tabatabaee et al., 2008 |  |  |  |  |  |  |  |  |
| Malla et al., 2020 |  |  |  |  |  |  |  |  |
| Chiliza et al., 2016 |  |  |  |  |  |  |  |  |
| Adhikari, 2014 |  |  |  |  |  |  |  |  |
| Iyer et al., 2022 |  |  |  |  |  |  |  |  |
| Rangaswamy et al., 2012 |  |  |  |  |  |  |  |  |
| Iyer et al., 2010 |  |  |  |  |  |  |  |  |

Clearly described Not properly described Not described

1. **Cross-sectional studies- STROBE checklist for cross-sectional studies**

| **Author** | **Study design** | **Study setting** | **Participants-Eligibility criteria** | **Variables** | **Data source** | **Bias** | **Study size** | **Quantitative variables** |
| --- | --- | --- | --- | --- | --- | --- | --- | --- |
| Mwesiga et al., 2021 |  |  |  |  |  |  |  |  |
| Ventura et al., 2021 |  |  |  |  |  |  |  |  |
| Iyer, Taksal, et al., 2022 |  |  |  |  |  |  |  |  |
| Mottaghipour et al., 2010 |  |  |  |  |  |  |  |  |
| Singh et al., 2023 |  |  |  |  |  |  |  |  |

Clearly described Not properly described Not described

**5- Qualitative study – JBI tool**

| **Author-** Vaitheswaran et al., 2021 | | |
| --- | --- | --- |
| **1.** | Congruity between the stated philosophical perspective and the research methodology |  |
| **2.** | Congruity between the research methodology and the research question or objectives |  |
| **3.** | Congruity between the research methodology and the methods used to collect data |  |
| **4** | Congruity between the research methodology and the representation and analysis of data |  |
| **5.** | Congruity between the research methodology and the interpretation of results |  |
| **6** | Statement locating the researcher culturally or theoretically. |  |
| **7** | The influence of the researcher on the research, and vice-versa, is addressed. |  |
| **8** | Participants, and their voices, are adequately represented. |  |
| **9** | Is the research ethical according to current criteria or, for recent studies, and is there evidence of ethical approval by an appropriate body? |  |
| **10** | The conclusions drawn in the research report flow from the analysis, or interpretation, of the data |  |

Clearly described Not properly described Not described

Lockwood C, Munn Z, Porritt K. Qualitative research synthesis: methodological guidance for systematic reviewers utilizing meta-aggregation. Int J Evid Based Healthc. 2015;13(3):179–187
