# Supplementary material for: Early Intervention in Psychosis and Management of First Episode Psychosis in Low- and Lower-Middle-Income Countries: A Systematic Review
Source: Schizophr Bull. 2024 Mar 25;50(3):521–32. doi: 10.1093/schbul/sbae025 (PMC11059814; doi:10.1093/schbul/sbae025)
Supplement: sbae025_suppl_Supplementary_Appendix_7 [file sbae025_suppl_supplementary_appendix_7.docx]

**Appendix 7**

**Table 2 - Description of the Interventional Studies**

| **Non-Pharmacological Interventions** | | |
| --- | --- | --- |
| **Author** | **Intervention description** | **Key findings (as relevant to review)** |
| (Sadath et al., 2017) | - Seven sessions of group intervention, 60-90 mins each - Service providers: trained psychiatric social worker - Follow up: 1 & 3-month post-intervention | - Expressed emotions (EE)-carers at one-month follow-up-   the intervention group demonstrated significantly more reduction (t = −2.806; p < 0.007).   - There were no group differences at a three-month follow-up. - Group intervention produces short-term benefits for a reduction in EE among the carers. |
| (Adhikari, 2014) | - One ECT was given under General anaesthesia. - Same antipsychotic drugs at the same dose were continued after ECT. - Follow up: 1-, 6- & 12-month post ECT | - With ECT, both psychopathology and daily functioning significantly improved (p=0.001and p= 0.003) - The quality of life increased, and the need for psychiatric medicines significantly decreased. |
| (Thomas et al., 2017) | - SMS reminder of the date and time of the patient’s first clinic appointment was sent 5 & 3 days before the scheduled clinic appointment. | - Participants who received an SMS were significantly less likely to miss their first clinic appointment (OR=.50). |
| **Pharmacological Interventions** | | |
| **Author** | **Intervention description** | **Key findings (as relevant to review)** |
| (Saddichha et al., 2008) | - Metabolic screening -baseline and at 6 weeks. - Drugs & mean dosage -Olanzapine (n=35): 16.5 ± 4.6 mg, Risperidone(n=33): 4.4 ± 1.2 mg, and Haloperidol (n=31):13.4 ± 3.6 mg for six weeks - Control group/healthy community sample (n=51) | - All the parameters under the study are adversely affected by both olanzapine and risperidone. - Olanzapine had a high prevalence of MetS (20–25%) when compared to risperidone (9–24%) and haloperidol (0–3%). |
| (Tabatabaee et al., 2008) | - Patients received antipsychotics for 5-7 weeks (mean dose: 7.3 mg/day for haloperidol or equivalent) - Antidepressants (selective serotonin reuptake inhibitors), mood stabilizers (lithium or sodium valproate) and ECT (n=4) administered if indicated. - Assessment of positive and negative symptoms and global assessment functioning was done on admission and at discharge (6 ±1weeks after admission) | - Good acute response to antipsychotic treatment (functional response rate 67.5%, Negative symptoms response rate 71.4% Positive symptoms response rate 91.5%) - If clinicians were given direction in identifying patients who had a decreased likelihood of responding to standard treatment, it could help those in the vulnerable group.   . |
| (Modabbernia et al., 2014) | - Patients with first-episode schizophrenia were randomly assigned to olanzapine plus either melatonin 3 mg/day or matched placebo for 8 weeks. - The patients were hospitalized for the first few weeks and then were followed up in the outpatient clinic. - Anthropometric and metabolic parameters and psychiatric symptoms were assessed using the Positive and Negative Syndrome Scale (PANSS) at baseline, at week 4, and at week 8. | - In patients with FEP, short-term melatonin therapy reduces weight gain, abdominal obesity, and possibly hypertriglyceridemia induced by olanzapine. - Signification reduction in the metabolic side effects- weight-p= 0.023, body mass index, kg/m2-p= 0.024, waist circumference, cm- p= 0.041 - Significant reduction in the Positive and Negative Syndrome Scale (PANSS) total score (p=0.014) |
| (Kaur et al., 2023) | - Patients were assessed for baseline severity of psychopathology. - Oral group received a daily dosage of oral haloperidol. - LAI group received LAI haloperidol decanoate fortnightly. - Doses were adjusted by the treating clinician. - Patients were assessed at weeks 2, 4, 6, 8, and 12. - Scales were applied to monitor response to treatment, side effects, compliance through Medication Possession Ratio (MPR and WHO quality of life. | - The LAI group demonstrated significantly better quality of life than the oral group (P = 0.023). - Patients on LAI (81%) on average had better adherence than those on oral haloperidol (77.4%) although a significant difference was present only during the initial assessment. - During the initial stages of therapy, a lesser number of side effects were reported with LAI compared to oral haloperidol. |
| **Both non-pharmacological and pharmacological studies on EIP** | | |
| **Author** | **Intervention description** | **Key findings (as relevant to review)** |
| (Malla et al., 2020) | - Case management, family intervention, and close monitoring of symptoms and social functioning. - Pharmacological management - All participants were prescribed a second-generation antipsychotic. - Positive & negative symptoms and family functioning were assessed up to two years. | - Both sites showed significant improvement in positive and negative symptoms over the 24 months. - The improvement was greater in Montreal for positive symptoms and in Chennai for negative symptoms. - Family support was higher in Chennai than in Montreal at month 3, month 12, and month 24. - Increasing family support and reducing treatment delay may lead to both early remission and improvements in longer-term outcomes across both contexts. |
| (Ventura et al., 2021) | - Help-seeking individuals were screened and assessed by the CHiRP Team provided CBT, cognitive rehabilitation training, supportive psychotherapy and medication management. - Six of the CHR positive were re-assessed at the three-month and six-month follow-up points. - CHR-negative received supportive psychotherapy and follow-up | - Feasibility of developing and implementing a CHiRP to identify and evaluate patients potentially at CHR in Tunisia. - One of the six patients transitioned to having schizophrenia at the 3-month follow-up assessment point. - highlight the need for early, targeted intervention that can be individualized for local cultural norms in CHR individuals |
| (Chiliza et al., 2016) | - Oral flupenthixol ,first flupenthixol decanoate dose to test for hyper-sensitivity. - Starting dose: 10mg with six weekly increments of 10mg to a maximum of 30 mg - The nurses provided psychoeducation. - Assessments- at baseline, weeks 1, 2, 4, and 6 and at months 3, 6, 9 and 12 | - Patients achieved response, 33 (19%) relapsed, 128 (62%) patients met remission criteria and 124 (60%) achieved full remission at 12 months. Ten (5%) participants met the criteria for treatment resistance. - Depot antipsychotic combined with an AMP is feasible, and may be an effective intervention in first-episode schizophrenia in resource-constrained settings |
| (Iyer et al., 2022) | - Both sites have open referral systems, provide free services, and follow a similar protocol. - Assessments- Socio-demographic data, clinical symptoms - Case management, family psychoeducation and other psychosocial interventions. - Medication adherence was recorded monthly based on information from patients and their families. - Duration -2 years | - Almost all Chennai patients retained contact with service providers throughout follow-up. - In Chennai, where the general rate of engagement was higher, at-distance contact was used significantly more than in Montreal,   where face-to-face contact predominated.   - Medication non-adherence was the strongest predictor of service disengagement. |
| (Rangaswamy et al., 2012) | - The intervention composed of medical and psychosocial components. - Pharmacological management included both first (haloperidol, chlorpromazine) and second generation (olanzapine, risperidone) antipsychotics. - All patients recruited into the program received the psychosocial interventions and home visits if required. - First 3 months of follow-up, these sessions were held twice a month and then gradually reduced to one session per month. - Duration -2 years | - There was significant improvement in positive and negative symptoms from baseline to 1st year with maximal improvement seen at 3 months after intake. - More women relapsed and more men dropped out. - Early intervention is possible even in settings that largely deal with chronic, sometimes never-treated psychotic patients. - Medication adherence in therapeutic engagement and psychosocial needs should be considered in the implementation of early intervention programs. |
