# Supplementary material for: Early Intervention in Psychosis and Management of First Episode Psychosis in Low- and Lower-Middle-Income Countries: A Systematic Review
Source: Schizophr Bull. 2024 Mar 25;50(3):521–32. doi: 10.1093/schbul/sbae025 (PMC11059814; doi:10.1093/schbul/sbae025)
Supplement: sbae025_suppl_Supplementary_Appendix_8 [file sbae025_suppl_supplementary_appendix_8.docx]

| **Appendix 8**  **Table 3 - Outcome measures of the included studies** | | | | |
| --- | --- | --- | --- | --- |
|  | **Author** | **Outcome of interest** | **Sample size** | **Outcome measures/key findings** |
| **Randomized Controlled Trials** | | | | |
| 1 | Thomas et al., 2017  Nigeria | Effectiveness of SMS reminders of clinic appointments among FEP patients | N=192  Intervention: N= 95  Control N:97 | The odds of attending the appointment in the intervention group [OR]=1.80, CI=1.02–3.19) |
| 2 | Saddichha et al., 2008  India | Effects of olanzapine, risperidone, and haloperidol on the development of metabolic syndrome in patients with first-episode schizophrenia | N=150  Intervention(I): N=99  Control (C): N=51 | Prevalence of MetS at the endpoint  *ATP III A criteria*  I = 10.1%, C= 2%; p= 0.005  *IDF criteria*  I=18.2%, C=0%; p=<0.001  Olanzapine (20–25%) risperidone (9–24%) haloperidol (0–3%) |
| 3 | Modabbernia et al., 2014  Iran | Efficacy of melatonin in the prevention of olanzapine-induced metabolic side-effects | N=48 Melatonin (I)N= 24  Placebo (C): N= 24 | Change from baseline at 8^th^ week -mean (SEM)  *(a)Weight kg-*  I=2.2 (0.60), C= 5.4 (1.22); p= 0.023  *(b)Body mass index, kg/m2-*  I=0.8 (0.21),C= 1.9 (0.40);p= 0.024  *(c)Waist circumference, cm-*  I= 1.1 (0.78), C=3.9 (1.09); p= 0.041 |
| 4 | Kaur et al., 2023  India | Effectiveness of long-acting injectable (LAI) antipsychotics for first‑episode schizophrenia over oral antipsychotics | N=72  LAI (I): N=38  Oral (C): N=34 | At the 12^th^ week (total)- mean (SD)  *(a)Change in PANSS score-*  I=28.5 (9.2), C=30.1(9.5); p=0.470  *(b)Quality of life on WHOQOL-BREF-*  I=105 (5), C=101 (7); p= 0.023 |
| **Non-randomized controlled trials** | | |  |  |
| 5 | Sadath et al., 2017  India | Expressed emotions & social support for carers of persons with FEP | N= 59  Intervention (I): N=38  TAU (C): N=21 | At one-month follow-up post-intervention  *(a)Overall expressed emotions (EE)-*  Reduction in the intervention group  (t = −2.806; p < 0.007)  (b) Social support subdomains –Improvement in the intervention group  “Significant others support”.  (F = 5.426; p = 0.0230)  “Friends support”  (F = 7.413; p = 0.009). |
| **Prospective studies** | | | | |
| 6 | Adhikari, 2014  Nepal | Efficacy of ECT in patients with first-episode schizophrenia (FES) | N=45  ECT received(I)=12  ECT not received(C) = 33 | Improvement -at 1^st^ year  *Psychopathology*  I= 22.58 (2.466), C= 26.48 (3.043); p=0.001  *Day-to-day functional status*  I= 87.08 (5.035),C= 79.64(7.420), p= 0.003 |
| 7 | Tabatabaee et al., 2008  Iran | Acute treatment response and its predictors in patients with first-episode psychosis | N=163 | *Predictors of functional response*  higher premorbid, lower baseline functioning, and acute onset  (x^2^ =30.093; df=3; p=<0.001; Nagelkerke R^2^=0.379) |
| 8 | Malla et al., 2020 | Clinical outcomes in FEP treated in Chennai (LMIC) and Montreal (HIC) using a similar EIS treatment protocol | Chennai: N=168  Canada: N= 165 | *Family support-*  (a)Chennai vs Montreal -mean (SD)  3^rd^ month-9.39(4.25) vs 8.31(3.91)  12^th^ month-10.64(3.72) vs 8.49(4.07)  24^th^ month-10.6(3.41) vs 8.79(3.46).  *(b)Reduction in symptoms (independent of family support)*  *Negative-*(F = 3.95 (d.f. = 1.49), P < 0.03  *Positive-* (F = 54.33 (d.f. = 1.51), P < 0.001  *(c)Duration of remission in months-*negative symptom remission Chennai vs Montreal -mean (SD)  16.10 (7.56) vs 9.78 (8.13) (t = −7.35, d.f. = 331, P < 0.001, Cohen’s d = −0.80). |
| 9 | Chiliza et al., 2016 | Feasibility and effectiveness of depot antipsychotic combined with an assertive monitoring program (AMP) in first-episode schizophrenia | N=207 | Comparison of scores at baseline and endpoint (12 months)  Positive and negative symptom scores  P= <0.0001  WHO Quality of Life-BREF Scale  P= <0.0001 |
| 10 | Iyer et al., 2022 | Differences in patients’ and families’ service engagement in similarly structured first-episode psychosis programs in Montreal, Canada, and Chennai | N=333 Chennai: N=168, Montreal: N=165 | PANSS total  Site effect- p= 0.05  Time effect-p=<0.001  Site x time effect-p= 0.009  Patient disengagement –  Montreal (N = 31/165, 19%)  Chennai (N = 2/168, 1%); p < 0.001  Family engagement-  Comparisons at baseline and months 6, 12, 18, and 24-significantly declined p < 0.001 |
| 11 | Rangaswamy et al., 2012 | The course and outcome of persons with the untreated first episode of psychosis, factors affecting the outcome, and DUP and its impact on outcome in psychosis. | N=47 | PANSS score & GAF- significant from inclusion to one year only- (t = 9.64, sig = .000) & (t = 8.005, sig = .000) not from year 1 to year 2.  DUP -less than 2 years had significantly better outcomes (x2 = 13.36, df = 1, sig = .001). |
| 12 | Iyer et al., 2010 | One year clinical and functional outcomes of first-episode psychosis program between Chennai & Montreal | India: N=61 Montreal: N= 88 | The dropout rate observed at the Indian site (5.4%) was considerably lower than at the Canadian site (18.95%) |
| **Observational-cross-sectional studies** | | | | |
| 13 | Singh et al., 2023  India | Comparison of pathways to care in first-episode psychosis (FEP) between North and South India, to inform solutions to bridge the treatment gap | N=177  South: N=72  North: N=105 | - AIIMS patients (North)were more likely to have a first encounter with a faith healer- p=0.003 and longer DUP (duration in weeks) (β = 17.68; p < .05) - In SCARF first contact with a faith healer was significantly associated with longer DUP p=<0.001 |
| 14 | Mwesiga et al., 2021  Uganda | Quality of the individual and group level interventions in first episode psychosis | N=156 | The proportion of the participants who received the component.  Individual psychoeducation-22.78% (n= 36), Multifamily group psychoeducation- 13.92% (n= 22), Group family psychoeducation-13.29%(n= 21) |
| **Evaluation of program** | | | | |
| 15 | Mottaghipour et al., 2010 | Effectiveness of training health professionals in adherence to protocol | 8 professionals | Adherence to the protocol-72%  Multiple-family group sessions-79%  Single-family home-based sessions -69% |
| **Qualitative study** | | | | |
| 16 | Vaitheswaran et al., 2021 | Challenges in FEP intervention program in a specialist mental health facility using the Consolidated Framework for Implementation Research (CFIR). | 27 (15 patients with FEP and 12 caregivers | CFIR successfully addressed implementation challenges evident in LMICs.  A service delivery model was proposed for first-episode psychosis in resource-limited settings. This model included a task-sharing approach. |
| **Tool development** | | | | |
| 17 | Iyer, Taksal, et al., 2022 | Development of patient-reported experience measures (PREMs) and family-reported experience measures (FREMs) for application in early psychosis services research | Chennai  Patient: N=29  Family: N=27  Montreal  Patient: N=31  Family: N=31 | Reliability  Cronbach's alphas -PREM & FREM at each site > 0.87.  Convergent validity  Overall, correlational values were significant p=<0.05 and in the predicted direction (patient and family versions at both sites) |
| **Feasibility Study** | | | | |
| 18 | Ventura et al., 2021 | Feasibility of developing a clinical high-risk program (CHiRP) program to identify youth at high clinical risk | N=10 | The results of this study revealed that a CHiRP could be developed and implemented in Tunisia and suggested the feasibility of employing the methods developed in high-income countries in LMIC settings. |
| PANSS-Positive and Negative Syndrome Scale for Schizophrenia, GAF-Global Assessment of Functioning, DUP- Duration of untreated psychosis, | | | | |
