# Supplementary material for: Early Intervention in Psychosis and Management of First Episode Psychosis in Low- and Lower-Middle-Income Countries: A Systematic Review
Source: Schizophr Bull. 2024 Mar 25;50(3):521–32. doi: 10.1093/schbul/sbae025 (PMC11059814; doi:10.1093/schbul/sbae025)
Supplement: sbae025_suppl_Supplementary_Appendix_9 [file sbae025_suppl_supplementary_appendix_9.docx]

**Appendix 9**

**Table 4: Characteristics of proposed EIP services in LMIC**

| **Author** | **Characteristics of EIS service** | **Recommendations/Proposed Service Outcomes** |
| --- | --- | --- |
| Iyer et al., 2010  India comparing with Canada | - Services are provided in outpatient, free of charge. - Referrals are taken from multiple sources, like hospitals, general practitioners, families/caregivers, and young people themselves. - Protocol includes case management, pharmacological management, family intervention, and close monitoring of symptoms and social functioning for at least two years. - Includes assertive follow-up model, an important component of specialized treatment for first-episode psychosis. | - Involvement of family members in treatment is a strong predictor of service engagement in specialized early intervention programs. |
| Singh et al., 2023  India | - Specialist mental health facility offering out-patient, in-patient, a day centre, and rehabilitation services. - The clinic provides case management, pharmacological management, family intervention, and close monitoring of symptoms & social functioning. | - The treatment gap could be bridged by a collaborative model between the faith healers and the current healthcare system. - This will promote integrated care and lessen the fragmentation of healthcare services. - Early intervention in psychosis services must be context-sensitive at a regional level rather than a national level in LMICs like India. |
| Mwesiga et al., 2021  Uganda | - Individual-level interventions   Pharmacotherapy (Selection of antipsychotic medication, mode of administration, dose, metabolic changes, monitoring medication side effects, steps to prevent weight gain and metabolic effects)/  Individual psychoeducation /Vocational and educational plans   - Group-level interventions   Multifamily group psychoeducation/ Group family psychoeducation | - Essential components for managing psychotic disorders are already available and could be implemented in resource-poor settings. - Quality of the available services needs to be improved. - More research is required on components, such as population-level interventions, which are essential to develop special early intervention services |
| Mottaghipour et al., 2010  Iran | - Family psychoeducation in the FEP program (multiple-family group education at a hospital or single-family home-based). - Training includes a 3-day- workshop followed by 12 supervision sessions | - Adequate training of health professionals to conduct family psychoeducational in the FEP program is crucial. - A hospital could be considered a better setting for delivering psychoeducational than a home setting. |
| Vaitheswaran et al., 2021    India | - Specialist FEP(SFEP) service is delivered by a team of psychiatrists and case managers. - Psychiatrists provide medical interventions, and the case managers coordinate care for individual patients and offer non-pharmacological interventions. - Some FEP individuals are managed by generic consultant psychiatrists and not referred to specialist FEP. | - Implementation barriers: cost, limited human resources, lack of formal training in FEP for healthcare workers, cultural and professional hierarchy, deviation from evidence-based guidelines, stigma in the community, and lack of funds for a sustainable program. - Implementation facilitators: leadership engagement, local and international collaboration experience, compatibility of the intervention with the existing system, recognition of the need for change, meeting the requirements of the service users, and rapport with service users. |
| Iyer et al., 2022  India & Canada | - Show me your care is, a new measure to explicitly evaluate the behaviors of treating teams, a construct related to, but separate from, user satisfaction | - By making real-time patient family-reported data about their treatment experience available, service providers will be better equipped to deliver high-quality care. - This has been developed in 3 languages and tested in both LMIC and high-income country settings. Thus, it has a potential for global application. |
